# Impact of a Culturally Tailored Educational Intervention on Human Papillomavirus Knowledge and Vaccination Intentions Among Women in Saudi Arabia: Quasi-Experimental Study

**DOI:** 10.2196/104529

**Published:** 2026-09-16

**Authors:** Ebtesam A Elsayed, Fathia Ahmed Mersal, Heba Ahmed Osman Mohamed, Ala M Aljehani, Najla M Aljehani

**Affiliations:** 1 Family and Community Health Nursing Department Faculty of Nursing Ain Shams University Cairo Egypt; 2 Public Health Department College of Health Sciences Saudi Electronic University Riyadh Saudi Arabia; 3 Public Health Nursing Department College of Nursing Northern Border University Arar Saudi Arabia; 4 Maternal and Child Health Nursing Department College of Nursing Northern Border University Arar Saudi Arabia; 5 Department of Pathology College of Medicine Imam Mohammad Ibn Saud Islamic University Riyadh Saudi Arabia

**Keywords:** culturally adapted intervention, HPV vaccination, HPV, human papillomavirus, IMB, information-motivation-behavioral skills, Saudi Arabia, sexually transmitted infection

## Abstract

**Background:**

Human papillomavirus (HPV) is one of the most common sexually transmitted infections worldwide. Despite the availability of effective vaccines, uptake remains suboptimal in many settings, including Saudi Arabia, where cultural sensitivities, limited awareness, and vaccine hesitancy have been documented. Educational interventions grounded in behavioral theory, such as the information-motivation-behavioral skills (IMB) model, can help address informational and motivational barriers to HPV vaccination.

**Objective:**

This study aimed to evaluate the impact of an educational intervention in improving HPV vaccination decision-making among women in Northern Saudi Arabia.

**Methods:**

A quasi-experimental design (pretest-posttest with a control group) was used among women attending primary health care centers in the Northern Border Region of Saudi Arabia. A total of 380 participants, 190 in the control group and 190 in the intervention group, completed surveys at baseline (T1), 1-week post intervention (T2), and a 3-month follow-up (T3), assessing HPV-related knowledge, health awareness, attitudes, and vaccination intentions. The educational intervention consisted of a culturally adapted, evidence-based, 45-minute interactive group session delivered in primary health care centers.

**Results:**

Study retention was excellent (380/389, 97.7%). At the 3-month follow-up, intervention participants demonstrated medium to large effect size improvements compared with controls: knowledge (adjusted *β*=0.84, 95% CI 0.53-1.15; *P*<.001; Cohen *d*=0.45, medium effect), health awareness (*β*=0.88, 95% CI 0.53-1.23; *P*<.001; *d*=0.44, medium effect), attitude (*β*=0.70, 95% CI 0.42-0.98; *P*<.001; *d*=0.38, medium effect), and vaccination intentions (*β*=0.86, 95% CI 0.60-1.12; *P*<.001; *d*=0.82, large effect). Exploratory subgroup analyses found no significant effect modification by age, education, or health literacy (all interaction *P*>.10), suggesting consistent intervention effects across demographic characteristics.

**Conclusions:**

This culturally tailored educational intervention was effective in improving HPV-related knowledge, attitudes, health awareness, and vaccination intentions among women in Northern Saudi Arabia. Given the well-documented intention-behavior gap in vaccination uptake, future research should incorporate verified vaccine initiation and completion outcomes, along with longer follow-up, to assess whether enhanced intentions translate into actual vaccine uptake.

## Introduction

### The Global Challenge of Human Papillomavirus as a Non-HIV Sexually Transmitted Infection in Women

Human papillomavirus (HPV) is the most prevalent non-HIV sexually transmitted infection (STI) globally, and most sexually active individuals will acquire HPV during their lifetime [1-3]. Among women, persistent infection with high-risk oncogenic HPV types is a necessary cause of cervical cancer. It contributes to a broader spectrum of anogenital and oropharyngeal cancers, creating a substantial preventable burden across the life course [3-5]. HPV vaccination offers a critical opportunity for primary prevention of an STI-related cancer, with high efficacy against vaccine-type infection and intraepithelial neoplasia when administered before exposure [5-7]. Among young adult women beyond routine childhood immunization schedules, HPV vaccination is often positioned as a preference-sensitive decision requiring individualized counseling, yet vaccine coverage remains insufficient in many settings [8,9].

Incomplete vaccine uptake reflects both individual-level barriers (knowledge, attitudes, and motivation) and structural barriers (access, cost, and provider recommendation). While system-level changes are essential, individual-level educational interventions remain necessary to address modifiable cognitive and motivational barriers, particularly in contexts where structural access exists but is underused [9,10]. Individual barriers include limited awareness and misinformation, perceived stigma associated with STI prevention, low confidence in vaccine safety, concerns about fertility and long-term harms, and the “intention-behavior gap,” the well-documented phenomenon in which individuals express willingness yet do not translate intention into action [11-13]. These factors are particularly relevant for women’s health in culturally conservative contexts where sexual health discussions carry social stigma.

### HPV Vaccination Challenges in Saudi Arabia: Epidemiological Context and Cultural Barriers

In Saudi Arabia, cervical cancer incidence is comparatively low but nonnegligible: the Information Centre on HPV and Cancer (ICO)/International Agency for Research on Cancer (IARC) country fact sheet [14] estimates approximately 358 new cases and approximately 179 deaths annually, with an age-standardized incidence rate (ASR) of approximately 2.8 per 100,000 women and ranking eighth among cancers in women overall and in those aged 15 to 44 years. Recent registry-based analyses also show regional variation, with Makkah consistently reporting the highest rates, followed by Riyadh and the Eastern Region, and a favorable shift toward earlier stage at diagnosis between 2005 and 2019 (increase in localized-stage diagnoses; decline in unknown stage), underscoring geographic and stage heterogeneity relevant to prevention strategies [15,16]. The Saudi Cancer Registry (under the National Cancer Center, Saudi Health Council) continues to publish periodic incidence reports and program updates, improving data quality for planning and evaluation [17].

Regarding vaccine policy and access, the Saudi Ministry of Health (MOH) health-education pages recommend HPV vaccination for adolescents (girls and boys) at ages 11 to 12 years (can start at age 9 years) and catch-up through age 26 years, aligning with international guidance; adults aged 27 to 45 years may benefit on a case-by-case basis. Since 2022, public communications from MOH and independent evaluations have described school-based campaigns introducing HPV vaccination for early adolescents, with media notices highlighting the importance of early vaccination and a multicohort school-based rollout reported in 2022 (with subsequent years’ activity). Importantly, official, centralized MOH fee schedules for HPV vaccine are not published; doses delivered within school-based/public programs are provided at no charge to families, whereas private-sector prices are not standardized or publicly listed by MOH/Saudi Food and Drug Authority (SFDA) [18].

Contemporary studies in Saudi Arabia consistently demonstrate low HPV vaccine uptake despite rising awareness. For example, a 2023-2024 national survey found that although 60.85% of Saudi women had heard of HPV, only 8.25% had received the vaccine, and more than 70% expressed willingness, highlighting a clear intention-behavior gap [19]. Similarly, recent cross-sectional research shows that Saudi women continue to report fear of side effects, doubts about vaccine effectiveness, and low positive perceptions, indicating persistent informational and motivational barriers to HPV prevention efforts [20]. Among younger populations, such as nursing and medical students, studies report poor HPV knowledge, limited understanding of screening, and low vaccination rates, with over 70% unvaccinated, despite relatively high awareness [21,22].

Although overall awareness has significantly increased following Saudi MoH campaigns, HPV-related behaviors remain suboptimal. A national comparison from 2022 to 2024 documented a rise in HPV awareness from 38.8% to 71.1%, yet cervical cancer screening uptake remained low, with only 25.8% of women reporting ever having a Pap smear [23]. Cultural and social dynamics further shape preventive behaviors: barriers such as perceived lack of necessity, abstinence-based reasoning, safety concerns, and misconceptions about who the vaccine is “appropriate” for continue to impede uptake [24]. In family-centered decision contexts, parental acceptance of HPV vaccination is strongly influenced by education level, health care worker status, and especially receiving a recommendation from the MoH or clinicians, demonstrating how social and institutional cues shape women’s access to preventive care [24-26].

Shared decision-making (SDM) principles may require adaptation in this context, as women’s health care decisions often involve family consultation and are influenced by clinician paternalism [27-29]. Educational interventions can support SDM by strengthening women’s knowledge and confidence to engage in vaccine discussions, ask questions, and express preferences to clinicians [30,31]. Therefore, this study aims to improve determinants of informed vaccination decisions, a foundational step toward SDM-compatible counseling.

### Educational Interventions, Theoretical Frameworks, and Evidence Gaps

Educational interventions have repeatedly demonstrated effectiveness in improving HPV-related knowledge, attitudes, and vaccine acceptance [11,32]. However, many interventions lack cultural tailoring and do not address context-specific concerns such as fertility misconceptions, stigma, and family influence, and they often fail to incorporate behavioral theory to explain how information converts to lasting motivation and action [11,33,34]. Ensuring equitable reach requires evaluating whether interventions maintain effectiveness across differing levels of health literacy, as literacy-related disparities substantially affect the ability to process health information and may reduce the intervention’s public health impact [35-37].

The information-motivation-behavioral skills (IMB) model proposes that behavior change is driven by accurate, actionable information, motivation (personal and social), and the behavioral skills needed to execute decisions, an especially relevant structure for vaccine uptake behaviors that require navigating appointments, consent, and follow-through [38,39]. IMB-based educational interventions have improved HPV vaccination-related outcomes in other contexts, including web-based education among female college students [40]. Yet, within Saudi Arabia and the broader Middle Eastern region, many studies remain cross-sectional, focus on university settings, or assess knowledge and hesitancy without testing scalable, culturally adapted, primary care–compatible intervention models [20,41]. Additionally, registry and international sources emphasize that although overall cervical cancer incidence is low, regional disparities and ongoing cases justify prevention efforts, particularly in high-population regions such as Makkah and Riyadh.

### Study Rationale, Objectives, and Research Questions

This study addresses these gaps directly by evaluating a culturally tailored educational intervention to improve HPV vaccination decision-making among women in Northern Saudi Arabia. This study makes 3 distinct contributions. First, it addresses HPV vaccination as both an informational challenge and an SDM implementation challenge, where women’s concerns, stigma, and family/social context must be addressed to support informed and values-concordant decisions [27,29,30]. Second, it applies a behavioral theory lens (IMB) to structure how education targets the specific mechanisms most plausibly linked to vaccine initiation behaviors [38,39]. Third, it focuses on a Saudi setting where published evidence indicates persistent gaps in knowledge and uptake and where culturally tailored prevention strategies are needed to reduce HPV-related disease burden among women [4,19,20]; contemporaneous national and international data further justify the focus, citing current case counts, regional variation, and active public health efforts.

### Research Questions

Research question 1 assesses whether a culturally tailored, IMB-based educational intervention improves HPV-related knowledge, health awareness, attitudes, and vaccination intentions among women in Northern Saudi Arabia compared to standard care. Research question 2 assesses whether intervention effects are sustained at 3-month follow-up. Research question 3 assesses whether intervention effects vary by age, education level, health literacy, or prior vaccination experience.

### Hypotheses

Hypothesis 1 states that intervention participants will demonstrate significantly greater improvements in all 4 outcome domains compared to controls at post-intervention and 3-month follow-up. Hypothesis 2 states that effects will be sustained (no significant decay) between postintervention and 3-month assessments. Hypothesis 3 states that intervention effects will not significantly differ across demographic subgroups (consistent effectiveness).

## Methods

### Study Design

A quasi-experimental design (pretest-posttest with a control group), with a pragmatic, nonrandomized allocation approach, was used due to operational constraints within the primary health care (PHC) system that precluded individual-level randomization, combined with concerns about contamination between intervention and control participants within the same clinic sessions. Outcomes were assessed at 3 time points: baseline (T1), approximately 1 week after the intervention session (T2), and 3 months after baseline (T3). The study is reported in accordance with the Transparent Reporting of Evaluations with Nonrandomized Designs (TREND) statement for nonrandomized evaluations of behavioral and public health interventions; a completed TREND checklist is provided in Multimedia Appendix 1. Causal language is used cautiously given the nonrandomized design, and effect estimates are interpreted as associations consistent with an intervention effect.

### Participants and Setting

#### Eligibility Criteria

Women who were currently pregnant were excluded because HPV vaccination is typically deferred until the postpartum period and because pregnancy-related clinical contexts could have introduced heterogeneity in decision urgency and confounded intervention effects. Additional exclusion criteria were (1) health care professionals or trainees likely to have specialized knowledge of HPV vaccination (eg, nursing, medical, or public health students or staff involved in health services), (2) current participation in another vaccine-related study, (3) cognitive impairment limiting informed consent or questionnaire completion, and (4) completion of the full HPV vaccination series. Numbers of women screened, excluded, and enrolled are reported in the Participant Flow and Retention section, in line with reporting standards for nonrandomized studies.

#### Recruitment and Enrollment

The study was implemented between December 2025 and May 2026 in 4 PHCs in the Northern Border Region, Saudi Arabia. Women attending the participating PHCs for routine services were approached by trained female research staff, screened for eligibility, and invited to participate; written informed consent was obtained from all enrolled women. Enrollment counts are reported in the Participant Flow and Retention section.

#### Allocation Procedure (Nonrandom)

Allocation was nonrandom and based on clinic scheduling/time of attendance to maximize feasibility and minimize disruption to routine PHC operations. Women attending designated morning clinic sessions at participating sites were invited to join the intervention arm, whereas women attending afternoon clinic sessions served as the control arm.

#### Threats to Internal Validity

Nonrandom, time-based allocation introduces potential selection bias, as timing of clinic attendance can correlate with employment status, caregiving responsibilities, health-seeking motivation, and socioeconomic factors. We prospectively collected and adjusted for key sociodemographic and health covariates (Outcome Measures section) to address measured confounding, but unmeasured factors associated with clinic attendance timing (eg, health motivation, urgency of care-seeking) could confound intervention effects. To quantify the magnitude of potential bias, standardized differences were calculated for all baseline covariates (Table 1), and sensitivity analyses were conducted (Statistical Analysis section). Although standardized differences <.10 suggested minimal observed imbalance across measured characteristics, causal inference remains limited, and estimates are interpreted as associations consistent with an intervention effect rather than as definitive causal effects. To minimize contamination, intervention and control sessions were scheduled on different days when feasible, and participants were asked not to share educational materials with other clinic attendees. Clinic staff provided routine care to both groups without knowledge of study arm allocation to the extent possible in this pragmatic trial. Participants and facilitators were not blinded to group allocation, which is inherent to the delivery of an educational intervention in a nonrandomized design. T3 telephone interviewers were partially blinded, as described in the Data Collection Procedures section.

**Table 1:** Baseline demographic characteristics and potential confounders. Data are presented as mean (SD) for continuous variables and n (%) for categorical variables.

Characteristic					Intervention group (n=190)			Control group (n=190)			*P* value^a^			Standardized difference^b^
Demographics														
	Age (years), mean (SD)			28.4 (6.8)			28.9 (7.1)			.47			.07	
	Age categories, n (%)										.85			.03
		18-26	82 (43.2)			79 (41.6)								
		27-35	78 (41.1)			81 (42.6)								
		36-45	30 (15.8)			30 (15.8)								
	Marital status, n (%)										.76			.03
		Single	94 (49.5)			91 (47.9)								
		Married	81 (42.6)			85 (44.7)								
		Divorced/widowed	15 (7.9)			14 (7.4)								
	Socioeconomic Factors													
	Education level, n (%)										.71			.05
		High school or less	38 (20.0)			42 (22.1)								
		University degree	126 (66.3)			121 (63.7)								
		Postgraduate	26 (13.7)			27 (14.2)								
	Employment status, n (%)										.68			.04
		Employed	112 (58.9)			108 (56.8)								
		Unemployed/student	78 (41.1)			82 (43.2)								
	Monthly household income (SAR^c^), n (%)										.89			.04
		<10,000	52 (27.4)			49 (25.8)								
		10,000-20,000	89 (46.8)			92 (48.4)								
		>20,000	49 (25.8)			49 (25.8)								
Health-related factors														
	Health literacy score (0-50), mean (SD)			34.2 (8.6)			33.8 (8.9)			.64			.05	
	Previous vaccination experience, n (%)			142 (74.7)			138 (72.6)			.65			.05	
	HPV^d^ vaccination history (partial), n (%)			8 (4.2)			6 (3.2)			.60			.06	
	Family history of cervical cancer, n (%)			18 (9.5)			21 (11.1)			.61			.05	
	Regular gynecological check-ups, n (%)			87 (45.8)			83 (43.7)			.69			.04	
Baseline outcome measures														
	Knowledge score (1-7; transformed)^e^, mean (SD)			2.97 (1.40)			2.94 (1.42)			.86			.02	
	Health awareness score (1-7), mean (SD)			3.05 (1.43)			2.93 (1.41)			.41			.08	
	Attitude score (1-7, transformed)^e^, mean (SD)			2.92 (1.43)			2.91 (1.40)			.92			.01	
	Vaccination intentions (1-7), mean (SD)			3.99 (1.04)			3.98 (1.08)			.94			.01	

^a^*P* values were derived from chi-square tests for categorical variables and 2-tailed independent-samples *t* tests for continuous variables.

^b^Standardized differences <.10 indicate minimal baseline imbalance.

^c^SAR 1=US $0.27 as of September 5, 2026.

^d^HPV: human papillomavirus.

^e^Knowledge and attitudes scores are linearly transformed from the original scales (0-9 and 1-5, respectively) to a 1-7 scale for comparability across outcomes.

#### Retention and Follow-Up

Participant flow, retention, and completeness of follow-up are reported in the Participant Flow and Retention section. Baseline characteristics were compared between participants who completed all assessments and those lost to follow-up; results of this comparison between participants who completed follow-up vs those who did not are provided in Multimedia Appendix 2. All 380 participants included in the analytic sample completed follow-up within the prespecified window; the final 3-month interview was completed on May 30, 2026, and no further follow-up assessments were undertaken after that date.

### Sample Size

Sample size was estimated using G*Power 3.1 for repeated-measures ANOVA with within-between interaction. The primary test was the *F* test of the group × time interaction in a 2 (group) × 3 (time) mixed design, for which the test statistic follows a noncentral *F* distribution with numerator *df* = (2 − 1)(3 − 1) = 2 and denominator *df* = 2(N − 2), with sphericity assumed (ε=1). Assuming a moderate effect size (*F*=0.25, equivalent to Cohen *d*≈0.50), an α of .05, power of 0.80, a total of 3 measurement time points, and a within-subject correlation of 0.5, the sample size required was 82 participants per group. Anticipating 20% attrition, we targeted 103 per group (n=206). Actual enrollment (n=389) substantially exceeded this target, providing adequate power for primary analyses.

Although the achieved sample provides sufficient power for main effects, the exploratory subgroup analyses (Statistical Analysis section) were not formally powered and should be interpreted as hypothesis-generating: interaction tests require approximately 4 times the sample size of main-effect tests, and with 190 participants per group, statistical power for detecting subgroup interactions was estimated at 40% to 50%.

### Intervention Development and Implementation

#### Theoretical Framework

The intervention was grounded in the IMB model [30], which proposes that health behavior adoption is driven by (1) accurate and accessible information, (2) motivation (personal and social), and (3) behavioral skills needed to translate decisions into action. The IMB model has been applied to sexual health behaviors and preventive decision-making, including STI prevention [42,43].

#### Patient and Public Involvement

##### Cultural Adaptation

A structured cultural adaptation framework consisting of 4 phases was used, developed with input from the target population and stakeholders (patient and public involvement): (1) information gathering through focus groups with 32 women from the target population and interviews with 15 stakeholders, including PHC clinicians (n=8), community health workers (n=4), and women’s health advocates (n=3), to identify context-specific barriers (eg, STI stigma, HPV/HIV confusion, fertility concerns, and preference for gender-concordant delivery); (2) preliminary adaptation and development of culturally congruent messaging and delivery strategies; (3) pilot testing with 20 women to refine clarity, acceptability, and session length, after which the session was reduced from 60 minutes to 45 minutes and terminology was revised to replace “sexually transmitted” with “virus that can cause cancer” in several sections to reduce stigma (pilot acceptability rating 4.6/5.0 for clarity and 4.8/5.0 for cultural appropriateness); and (4) refinement and finalization of materials and delivery protocols.

All intervention materials (session scripts, facilitator guides, and the Arabic pamphlet) were developed in Arabic by the bilingual study team and back-translated into English to verify content fidelity; discrepancies identified during back-translation were resolved by consensus before pilot testing.

##### Intervention Content and Delivery

The intervention consisted of a 45-minute interactive group session delivered in private rooms within PHCs to groups of 10 to 15 participants, facilitated by 2 trained female health educators.

Content was standardized and organized into four modules aligned with IMB constructs: (1) understanding HPV and cervical cancer (information; 15 minutes); (2) HPV vaccines: safety, efficacy, and access (information; 15 minutes); (3) making an informed vaccination decision (motivation and behavioral skills; 10 minutes); and (4) addressing misconceptions (information and motivation; 5 minutes). Key misconceptions explicitly addressed included confusion between HPV and HIV; beliefs that vaccination causes infertility; concerns that vaccination is not permissible (Halal); and the misunderstanding that HPV only affects sexually promiscuous individuals. Key cultural adaptations included framing HPV primarily as a cancer-preventing vaccination to reduce STI-related stigma, ensuring female-only facilitation, clarifying differences between HPV and HIV, addressing common fertility concerns, and explaining HPV risk in married and monogamous relationships.

Participants received an 8-page Arabic pamphlet written at approximately a sixth-grade reading level (assessed using the Arabic adaptation of the Simple Measure of Gobbledygook [SMOG] readability formula) summarizing key points. Complete session scripts, facilitator guides, and educational materials are provided in Multimedia Appendix 3.

##### Facilitator Training and Fidelity Monitoring

Educators completed a standardized 2-day training program (16 hours) covering IMB principles, HPV vaccine content, communication skills for sensitive topics, and fidelity procedures. To assess fidelity, 20% (8/40) of sessions were audio-recorded with additional participant consent and independently coded by 2 trained raters using a 15-item fidelity checklist. Interrater reliability for fidelity coding was substantial (Cohen κ=0.78, 95% CI 0.69-0.87). Mean fidelity was 93% (range 87%-100%), indicating high adherence. Sessions with fidelity <90% (n=3) triggered facilitator feedback and retraining.

##### Control Condition

Control participants received standard PHC care without structured HPV education. Controls completed the same outcome assessments at T1 to T3. After completion of the 3-month follow-up, control participants were offered delayed access to the educational session (148/190, 78% accepted).

### Outcome Measures

#### Instrument Development and Translation

A structured questionnaire was developed by adapting items from previously published HPV knowledge/attitude instruments [44,45] and tailoring language for cultural appropriateness. The instrument underwent forward-backward translation (English-Arabic), expert panel review (n=5 content experts), and cognitive interviewing (n=8 women from the target population). Pilot testing (n=20) provided preliminary support for internal consistency (Cronbach α≥0.78) and 2-week test-retest stability (intraclass correlation coefficient [ICC] ≥0.76). While these values suggest acceptable measurement properties, the small pilot sample and lack of comprehensive validation (eg, confirmatory factor analysis and convergent/discriminant validity testing) preclude definitive conclusions about scale validity. We therefore interpret the adapted instruments as research tools requiring further validation in Saudi populations.

#### HPV Knowledge (Coprimary Outcome)

HPV knowledge was assessed using 9 “true,” “false,” or “do not know” items covering HPV transmission, cancer risk, vaccine efficacy, and safety. Items were scored 1 for correct and 0 for incorrect or “do not know” responses (range 0-9). Because items were dichotomously scored, internal consistency was estimated using the Kuder-Richardson formula 20 (KR-20), which is mathematically equivalent to Cronbach α for binary items; the reported value (KR-20=0.78) refers to this estimate. “Do not know” responses were coded as incorrect by a priori specification, consistent with the interpretation that they do not reflect accurate knowledge. For comparability across outcomes, raw knowledge scores were linearly transformed to a 1 to 7 scale using the formula: transformed score = 1 + (raw score / 9) × 6; with higher scores indicating greater knowledge.

#### HPV-Related Health Awareness (Coprimary Outcome)

HPV-related health awareness was measured using 5 items assessing awareness of personal risk, disease burden, and cancer prevention on 7-point Likert scales (1=not at all aware to 7=extremely aware). The mean score was computed, with higher scores indicating greater awareness (Cronbach α=0.81).

#### Attitudes Toward HPV Vaccination (Coprimary Outcome)

Attitudes toward HPV vaccination were measured using 8 items assessing beliefs about vaccine safety, effectiveness, necessity, and appropriateness on Likert scales (1=strongly disagree to 7=strongly agree). The mean score was computed, with higher scores indicating more favorable attitudes (Cronbach α=0.84). Raw attitude scores (range 1-5) were linearly transformed to the common 1 to 7 metric using the formula: transformed score = 1 + (raw score / 5) × 6.

#### HPV Vaccination Intentions (Coprimary Outcome)

HPV vaccination intentions were measured using 5 items assessing likelihood of vaccine uptake behaviors (seeking information, discussing with family, and scheduling vaccination) on 7-point likelihood scales (1=extremely unlikely to 7=extremely likely). The mean score was computed, with higher scores indicating stronger intentions (Cronbach α=0.88).

#### Justification for Score Transformation

Because the 4 outcomes were measured on different raw scales, all scores were placed on a common 1 to 7 metric to permit direct comparability of effect sizes across outcomes. Linear transformation preserves within-outcome rank order and does not alter inferential statistics; all primary models were reestimated using original-scale scores, with identical conclusions**.** All outcome items and scoring procedures are reported in Multimedia Appendix 4.

### Data Collection Procedures

At baseline (T1), participants provided written informed consent and completed the Arabic questionnaire in a private PHC room (approximately 20 to 25 minutes). Trained female research assistants were available to clarify procedural questions without guiding responses.

At T2, a mean of 6.8 (SD 1.2) days after the intervention session (approximately 1 week after baseline), both groups completed the same questionnaire using identical paper-based, in-person administration procedures.

At T3, approximately 3 months postbaseline (mean 91.4, SD 8.6 days), follow-up data were collected via standardized telephone interviews conducted by trained female staff. T3 telephone interviewers were partially blinded: they did not know participant group allocation at the time of the call but could potentially infer group status if participants spontaneously mentioned attending an education session. To minimize detection bias, interviewers used scripted questions, avoided unstructured conversation, and were trained to redirect discussion to assessment items if participants volunteered unsolicited information about study participation.

To assess potential mode effects (in-person paper vs telephone), we conducted sensitivity analyses comparing T2 (paper) vs T3 (telephone) response patterns within the control group, which received no intervention between these time points. No significant differences were observed (all *P*>.15), suggesting minimal mode-related bias (Multimedia Appendix 5).

### Statistical Analysis

All statistical analyses were performed using R software (version 4.3.1; R Core Team), following a prespecified analysis plan.

#### Multiple Testing Considerations

We specified 4 coprimary outcomes to comprehensively assess intervention effects across the IMB model’s theoretical domains (information, motivation, and behavioral intention). Because these outcomes are conceptually and empirically correlated (*r*=0.42-0.68 at baseline) and represent a unified construct (vaccine readiness), we did not apply multiple testing corrections for primary analyses of time × group effects, consistent with recommendations for evaluating conceptually linked outcomes. However, for exploratory subgroup analyses involving 4 moderators tested across 4 outcomes, we applied Bonferroni correction (α=.05/4=.0125 per outcome domain, testing each moderator separately).

#### Descriptive and Baseline Balance Analyses

Baseline characteristics were summarized using mean (SD) for continuous variables and n (%) for categorical variables. Between-group balance was assessed using 2-tailed independent-samples *t* tests, chi-square tests, and standardized differences (Cohen *d* for continuous variables; Cramer V for categorical variables), with standardized differences <.10 interpreted as excellent balance and indicative of minimal confounding from measured covariates.

#### Primary Models

For each outcome, linear mixed-effects models with participant-level random intercepts were fitted to account for repeated measures, including fixed effects for time (T1, T2, and T3), group (intervention vs control), and the time × group interaction. Three nested models were estimated: model 0 (unadjusted); model 1 (adjusted for age, education, and marital status); and model 2, the primary model (additionally adjusted for health literacy, previous vaccination experience, and family history of cervical cancer). The time × group interaction at T2 and T3 represents the primary intervention effect. Effect sizes were calculated as Cohen *d* using pooled change-score standard deviations (T3 − T1) as the primary metric, appropriate for repeated-measures designs**;** for comparability with previous studies, Cohen *d* using pooled baseline SDs is also reported in the tables.

#### Secondary and Robustness Analyses

We computed within-group change scores (T3 − T1) and between-group differences in change with 95% CIs to provide interpretable estimates alongside model-based results.

Exploratory subgroup analyses tested effect modification by age (18-26 years vs 27-45 years; cutoff reflects typical transition from younger adult to family-building life stages), education (university vs below university level), health literacy (inadequate/marginal vs adequate) using validated Brief Health Literacy Screen cutoffs [46], and prior vaccination experience (any vs none) using 3-way interaction terms (time × group × moderator). Each moderator was tested separately with Bonferroni-adjusted α=.0125 per outcome domain.

Sensitivity analyses included intention-to-treat using last observation carried forward (LOCF), per-protocol analysis (participants attending ≥80% of session content), multiple imputation (50 datasets; multivariate imputation by chained equations [MICE]), complete-case analysis (primary), alternative covariance structures (unstructured and autoregressive), and assessment of potential administration mode effects (telephone vs paper). Because the intervention was delivered in groups across multiple clinics and facilitators, sensitivity models additionally included random intercepts for clinic (4 levels) and intervention session (40 levels), with cluster-robust (sandwich) SEs; ICCs are reported in Multimedia Appendix 6. These analyses addressed the potential clustering of responses within PHCs and intervention sessions**.**

Results from all sensitivity analyses are reported in Multimedia Appendix 6. Model assumptions were evaluated via residual diagnostics, quantile-quantile (Q-Q) plots, and variance inflation factors (VIF<2.5). Models were fitted using restricted maximum-likelihood with the lme4 package. Reporting followed the TREND statement for nonrandomized evaluations of behavioral and public health interventions [47]; a completed TREND checklist is provided in Multimedia Appendix 1.

### Ethical Considerations

Ethical approval for this study was granted by the Local Committee of Bioethics at Northern Border University (HAP-09-A-043; decision number 90/25/H). All participants were informed about the study objectives and procedures, and written informed consent was obtained before participation. The research team adhered strictly to all bioethics guidelines stipulated by the committee throughout the study.

Consent procedures emphasized voluntariness, confidentiality, the right to withdraw, and the possibility of discomfort when discussing sensitive topics. Consent forms were written in Arabic at approximately a sixth-grade reading level (assessed using the Arabic SMOG formula) and reviewed orally by female staff to ensure comprehension.

Confidentiality was maintained using unique IDs, separate secure storage of identifiers and data, encrypted password-protected databases, and staff confidentiality training. Data will be retained for 5 years and then securely destroyed by institutional policy.

Risk mitigation strategies included female-only delivery teams, private spaces, flexible scheduling, permission to skip any question, and cancer-prevention framing to minimize stigma. Adverse events were monitored through participant self-report at each assessment and facilitator incident reports. No adverse events were reported. Control participants were offered delayed access to the intervention after completion of the 3-month follow-up (148/190, 78% accepted).

This nonrandomized interventional study was not registered in a public trials registry because trial registration is not a requirement for quasi-experimental evaluations under applicable journal policy; the analysis plan was prespecified in writing before data analysis. Study methods and procedures were reviewed and approved by the institutional bioethics committee; no protocol amendments were made after data collection began.

## Results

### Participant Flow and Retention

A total of 512 women were assessed for eligibility between December 2025 and May 2026 in the 4 participating PHCs. Of these, 123 (24%) were excluded: 69 did not meet inclusion criteria (18 were currently pregnant, 28 were health care professionals or trainees with specialized knowledge, 4 had cognitive impairment limiting informed consent, and 19 had previously completed the full HPV vaccination series), 32 declined to participate, and 22 were excluded for other reasons (eg, planned relocation or inability to attend scheduled sessions). The remaining 389 women (76% of those screened) were enrolled and allocated to the educational intervention group (n=194) or the control group (n=195; Figure 1). All 380 participants included in the analytic sample completed the 3-month follow-up; the final follow-up assessment was completed on May 30, 2026.

In the intervention group, 99% (192/194) of enrolled participants received the educational intervention; 2 participants withdrew before the intervention session because of scheduling conflicts. In the control group, 97.9% (191/195) of enrolled participants completed the baseline assessment and remained in the study; 4 participants withdrew before follow-up assessments could begin (2 because of relocation and 2 because they could not be reached). During follow-up, an additional 2 participants from the intervention group and 1 from the control group were lost to follow-up (lost contact or declined further assessment). Consequently, 190 participants per group (380/389, 97.7%) completed all 3 assessments and constituted the analytic sample (Figure 1). Baseline characteristics did not differ significantly between completers and those lost to follow-up (all *P*>.40), suggesting minimal attrition bias.

**Figure 1:**
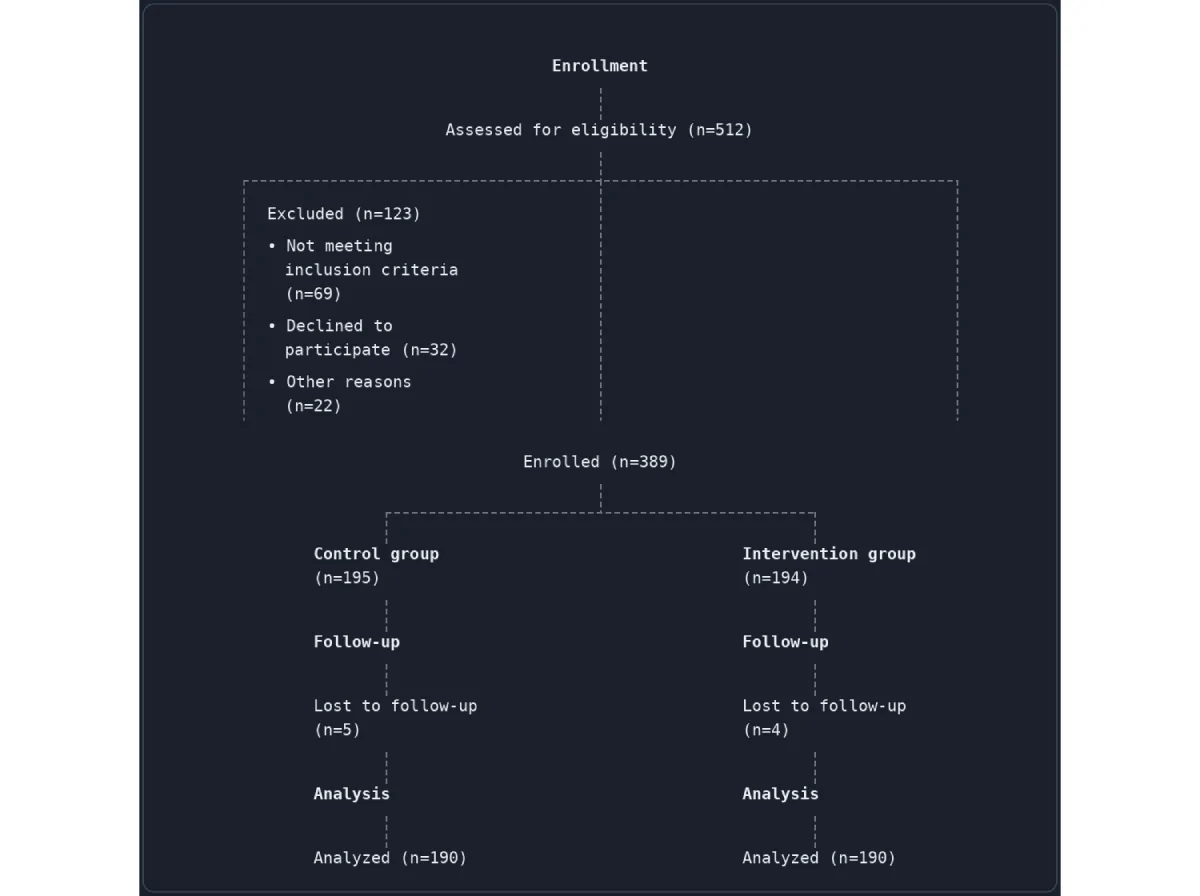
Flow diagram of participant enrollment, allocation, follow-up, and analysis.

### Baseline Characteristics and Balance Assessment

Baseline demographic, socioeconomic, and health-related characteristics were well-balanced between the study arms (Table 1). The mean age of participants was approximately 28.6 (SD 6.9) years, with the majority holding a university degree (n/N, 65%). Approximately 43% (n/N) of participants were married. Importantly, potential confounders such as health literacy, previous vaccination experience, and family history of cervical cancer showed no statistically significant differences between groups (all *P*>.05). Standardized differences for all covariates were less than 0.10, indicating minimal observed baseline imbalance despite nonrandom allocation and supporting comparability of groups for adjusted analyses.

Baseline outcome scores were also well-balanced: mean knowledge scores were 2.97 (intervention) vs 2.94 (control), health awareness 3.05 vs 2.93, attitude 2.92 vs 2.91, and vaccination intentions 3.99 vs 3.98 (all *P*>.40, standardized differences <.10). This balance across both baseline characteristics and outcome measures strengthens causal inference despite the nonrandomized design.

### Primary Outcome Analyses

#### HPV Vaccine Knowledge

The intervention was associated with significant and sustained improvements in HPV vaccine knowledge at 3 months (Table 2). While baseline scores were comparable (intervention: 2.97, 95% CI 2.77-3.17; control: 2.94, 95% CI 2.74-3.14; *P*=.86), the intervention group increased to 3.47 (95% CI 3.29-3.65) at T2 and to 3.74 (95% CI 3.57-3.91) at T3; the control group remained essentially unchanged (T2: 3.09, 95% CI 2.90-3.28; T3: 2.87, 95% CI 2.69-3.05). Between-group comparisons were statistically significant at T2 (*P*=.01) and T3 (*P*<.001). Effect sizes at T3 were Cohen *d*=0.58 using pooled baseline SD and Cohen *d*=0.45 using pooled change-score SD.

**Table 2:** Multivariable adjusted analysis of intervention effects on primary outcomes.

Outcome and time point		Unadjusted *β* (95% CI)	*P* value	Adjusted *β*^a^ (95% CI)	*P* value	Fully adjusted *β*^b^ (95% CI)	*P* value	*R* ^2^
Knowledge								
	Postintervention	0.38 (0.08-0.68)	.01	0.36 (0.07-0.65)	.02	0.35 (0.06-0.64)	.02	0.14
	3-month follow-up	0.87 (0.57-1.17)	<.001	0.85 (0.55-1.15)	<.001	0.84 (0.53-1.15)	<.001	0.21
Awareness								
	Postintervention	0.52 (0.17-0.87)	.004	0.50 (0.15-0.85)	.005	0.49 (0.14-0.84)	.006	0.16
	3-month follow-up	0.91 (0.56-1.26)	<.001	0.90 (0.55-1.25)	<.001	0.88 (0.53-1.23)	<.001	0.24
Attitudes								
	Postintervention	0.40 (0.12-0.68)	.006	0.38 (0.10-0.66)	.008	0.38 (0.09-0.67)	.009	0.12
	3-month follow-up	0.73 (0.45-1.01)	<.001	0.71 (0.43-0.99)	<.001	0.70 (0.42-0.98)	<.001	0.19
Intentions								
	Postintervention	0.50 (0.24-0.76)	<.001	0.49 (0.23-0.75)	<.001	0.48 (0.21-0.75)	<.001	0.18
	3-month follow-up	0.89 (0.63-1.15)	<.001	0.87 (0.61-1.13)	<.001	0.86 (0.60-1.12)	<.001	0.28

^a^Adjusted for age, education, and marital status.

^b^Additionally adjusted for health literacy, previous vaccination experience, and family history of cervical cancer.

#### HPV-Related Health Awareness

Awareness of HPV health risks increased significantly within the intervention arm (T1: 3.05, 95% CI 2.84-3.26; T2: 3.47, 95% CI 3.27-3.67; T3: 3.92, 95% CI 3.73-4.11), whereas the control group remained stable (T1: 2.93, 95% CI 2.73-3.13; T3: 3.01, 95% CI 2.81-3.21). Between-group differences were statistically significant at T2 (*P*=.004) and T3 (*P*<.001), with effect sizes of *d*=0.35 (baseline SD) at T2 and *d*=0.63 (baseline SD) at T3 (change-score *d*=0.44 in Table 3).

**Table 3:** Analysis of immediate postintervention effects: comparison of change scores.

Comparison and variable		Intervention Δ^a^, mean (SD)	Control Δ^a^, mean (SD)	Difference in change	*t* test (*df*)	*P* value	Cohen *d* (95% CI)^b^
Comparison of change scores (T2 – T1)							
	Knowledge	0.50 (1.92)	0.15 (2.00)	0.36	1.77 (378)	.08	0.18 (–0.02 to 0.38)
	Awareness	0.43 (1.86)	0.03 (2.04)	0.40	2.00 (378)	.05	0.21 (0.00 to 0.41)
	Attitude	0.44 (1.88)	0.06 (2.02)	0.38	1.92 (378)	.06	0.20 (–0.01 to 0.40)
	Intentions	0.66 (1.52)	0.17 (1.36)	0.49	3.31 (378)	.001	0.34 (0.14 to 0.54)
Comparison of change scores (T3 – T1)							
	Knowledge	0.77 (1.72)	–0.07 (1.81)	0.84	4.69 (378)	<.001	0.45 (0.25 to 0.65)
	Awareness	0.87 (1.69)	0.08 (1.88)	0.79	4.28 (378)	<.001	0.44 (0.24 to 0.64)
	Attitude	0.82 (1.74)	0.10 (1.95)	0.72	3.80 (378)	<.001	0.38 (0.18 to 0.58)
	Intentions	1.05 (1.28)	0.17 (1.24)	0.88	6.74 (378)	<.001	0.82 (0.62 to 1.02)

^a^Δ indicates the within-group change score.

^b^Cohen *d* was calculated using the pooled change score SD.

#### Attitudes Toward HPV Vaccination

Attitudes improved within the intervention arm (T1: 2.92, 95% CI 2.71-3.13; T3: 3.74, 95% CI 3.55-3.93), whereas control attitudes remained stable (T1: 2.91, 95% CI 2.71-3.11; T3: 3.01, 95% CI 2.81-3.21). Between-group analyses confirmed significantly more favorable attitudes in the intervention group at T2 (*P*=.006) and T3 (*P*<.001), with effect sizes of *d*=0.27 (baseline SD) and *d*=0.52 (baseline SD), respectively (change-score *d*=0.38 in Table 3).

#### Vaccination Intentions

The largest effects were observed for vaccination intentions. The intervention group showed a substantial increase from T1 (3.99, 95% CI 3.84-4.14) to T2 (4.65, 95% CI 4.49-4.81) and T3 (5.04, 95% CI 4.89-5.19). The control group showed small increases (T1: 3.98, 95% CI 3.82-4.14; T3: 4.15, 95% CI 3.99-4.31). Between-group comparisons indicated significantly stronger vaccination intentions in the intervention group at both T2 and T3 (both *P*<.001), with effect sizes of *d*=0.48 and *d*=0.71 (baseline SD), respectively, and *d*=0.82 using change-score SD at T3 (Table 3).

#### Summary of Effect Sizes

Across outcomes, intervention effects using pooled baseline SD ranged from *d*=0.27 to *d*=0.71 at follow-up. Effect sizes calculated using the prespecified primary metric (pooled change-score SD) ranged from *d*=0.38 to *d*=0.82 at follow-up (Table 3), consistent with medium-to-large effects.

### Adjusted Analyses Controlling for Confounders

Linear mixed-effects models were fitted for all primary outcomes to address potential baseline imbalance and confounding (Table 2). Three nested models were compared: model 0 (unadjusted), model 1 (adjusted for age, education, and marital status), and model 2, the primary model (additionally adjusted for health literacy, previous vaccination experience, and family history of cervical cancer).

The intervention effect (time × group interaction) was statistically significant across all adjusted models and across all 4 outcomes at T2 and T3. Adjustment for covariates produced minimal attenuation of effect estimates (*β* coefficients changed by <0.03 across models), indicating strong baseline balance and little measured confounding. Model *R*^2^ values ranged from 0.12 to 0.28, with the fully adjusted models providing the best fit (Multimedia Appendix 6). Model diagnostics confirmed the adequacy of the linear mixed models: residual plots showed no systematic patterns; Q-Q plots indicated approximately normal residuals; and all VIFs were <2.5.

### Cluster-Adjusted Sensitivity Analyses

Because the intervention was delivered in groups (10-15 participants) across multiple sessions (n=40), clinics (n=4), and facilitators (n=8), clustering of responses could have occurred. To address this concern (raised in BZ Major Comment 2), sensitivity models were refitted with random intercepts for clinic and intervention sessions, and with cluster-robust (sandwich) standard errors applied to the primary complete-case models. ICCs were modest at clinic and session levels (range 0.01-0.05). The cluster-adjusted models produced slightly larger point estimates and slightly wider confidence intervals than the primary models (maximum difference in *β* of ±0.04 for any outcome or time point), and all previously significant effects remained significant (*P*<.01). These analyses indicate that clustering does not materially alter inference for the primary outcomes.

### Change Score Analysis and Effect Sizes

To quantify the specific magnitude of the intervention effect, within-group change scores were calculated and compared directly between groups. Table 3 presents immediate postintervention changes (T2 – T1) as well as sustained changes at 3-month follow-up (T3 – T1).

At the immediate postintervention assessment, the intervention group achieved significantly greater gains than controls for health awareness (*P*=.05; *d*=0.21) and vaccination intentions (*P*=.001; *d*=0.34). Knowledge and attitudes showed positive trends that did not reach statistical significance at this early time point. However, by the 3-month follow-up, all 4 outcomes demonstrated significant between-group differences in change scores, with effect sizes ranging from *d*=0.45 (knowledge) to *d*=0.82 (intentions) using the change score pooled SD. The progressive strengthening of effects from postintervention to follow-up suggests sustained engagement with intervention content and potential social diffusion or reinforcement of learning over time.

### Subgroup Analyses and Effect Modification

Exploratory subgroup analyses were conducted to determine whether intervention effects varied by key demographic characteristics. We assessed interactions between the intervention and 4 prespecified moderators: age (18-26 years vs 27-45 years), education level (university vs below university level), health literacy (inadequate/marginal vs adequate using validated cutoffs), and previous vaccination experience (any vs none). Moderator effects were evaluated using 3-way interaction terms (time × group × moderator) in the mixed-effects models. Because time (2 levels), group (2 levels), and each moderator (2 levels) were binary, each interaction term has 1 numerator degree of freedom; denominator degrees of freedom were estimated using the Satterthwaite approximation. Using a Bonferroni-corrected significance level of α=.0125 per outcome domain (to account for testing 4 moderators separately), no significant 3-way interactions (time × group × moderator) were detected for any outcome.

For vaccination intentions (the outcome with strongest main effects), the interaction tests were: age (*F*_1,424.31_=1.23; *P*=.28), education (*F*_1,421.05_=0.87; *P*=.42), health literacy (*F*_1,418.52_=1.41; *P*=.23), and prior vaccination experience (*F*_1,425.14_=0.65; *P*=.52). Similar nonsignificant patterns were observed for other outcomes (Multimedia Appendix 6 provides complete stratified results and interaction statistics).

Point estimates suggested numerically larger gains for younger women (age 18-26 years: mean 3-month change +1.10, 95% CI 0.89-1.31 vs age 27-45 years: mean 3-month change +0.98, 95% CI 0.72-1.24), university-educated participants (+1.15, 95% CI 0.96-1.34 vs below university level: +0.92, 95% CI 0.65-1.19), and those with adequate health literacy (+1.18, 95% CI 0.99-1.37 vs inadequate/marginal: +0.89, 95% CI 0.64-1.14), but these differences were not statistically significant after multiplicity correction. The study was not formally powered for interaction tests (estimated power approximately 40%-50%); therefore, the absence of significant interactions should not be interpreted as evidence of equal effect magnitudes across subgroups, and these analyses are best treated as hypothesis-generating.

### Control Group Changes and Alternative Explanations

The control group also showed small increases across outcomes, particularly for vaccination intentions (from 3.98 at T1 to 4.15 at T3). Possible explanations discussed transparently include secular trends from concurrent national HPV awareness campaigns in Saudi Arabia, repeated measurement effects (familiarity with questionnaire items), and nonspecific attention during routine clinic visits within the time period. Although these changes were small and did not reach statistical significance in isolation, the primary inference is based on adjusted between-group comparisons that account for any incremental control arm change.

### Sensitivity Analyses

Comprehensive sensitivity analyses confirmed the robustness of primary findings across alternative analytic approaches (see Multimedia Appendix 4 for complete results). Intention-to-treat analysis using LOCF for the 9 participants lost to follow-up yielded effect estimates within 0.02 to 0.04 points of primary complete-case results, with all previously significant effects remaining significant (all *P*<.01).

Per-protocol analysis restricted to participants with 80% or greater session attendance (n=352; 92.7% of analytical sample) showed slightly stronger intervention effects, with *β* coefficients 5% to 8% larger than primary models (eg, vaccination intentions at 3 months: *β*=0.93, 95% CI 0.66-1.20 vs primary *β*=0.86). This dose-response pattern is consistent with greater engagement producing larger effects.

Multiple imputation with 50 datasets using MICE produced point estimates within 0.01 to 0.03 points of complete-case analysis, with nearly identical CIs and *P* values. Alternative covariance structures (unstructured and first-order autoregressive) yielded substantively identical conclusions (maximum difference in *β*=0.02; all previously significant effects remained *P*<.001).

Assessment of potential mode effects (in-person paper administration vs telephone) revealed no systematic bias. Within the control group (which received no intervention between T2 and T3), responses at T2 (paper) and T3 (telephone) showed no significant differences across all 4 outcomes (all *P*>.15; mean absolute difference <.08 points; Multimedia Appendix 5). This provides reassurance that the T3 telephone data collection mode did not introduce measurement artifacts. The consistency of findings across these diverse sensitivity analyses reinforces the internal validity and robustness of the trial results.

## Discussion

### Overview

This study demonstrates that a culturally tailored, IMB-based educational intervention significantly improved HPV-related knowledge, health awareness, attitudes, and vaccination intentions among women aged 18 to 45 years attending PHC centers in Northern Saudi Arabia. At 3-month follow-up, intervention participants showed medium-to-large effect-size improvements across all outcomes compared with controls, with effects sustained from the immediate postintervention assessments. These findings contribute to the limited evidence base on culturally tailored HPV education interventions in Middle Eastern contexts and provide the first controlled evaluation of an IMB-based approach delivered in Saudi primary care settings, but because allocation was nonrandom and based on clinic session timing, the present results estimate intervention association rather than a strict causal effect.

### Principal Findings and International Contextualization

The intervention yielded a substantial rise in vaccination intention, from 3.99 at baseline to 5.04 at the 3-month follow-up, representing a 15% absolute increase on a 7-point scale and constituting the largest observed effect, a pattern consistent with prior educational interventions. Similar gains have been documented among pregnant women in Saudi Arabia, where structured educational sessions produced twofold to threefold increases in HPV-related awareness [48]. Comparable outcomes were also observed among Tunisian female students, who demonstrated significant knowledge improvement following brief video-based instruction, with mean scores rising from 4.23 preintervention to 6.16 postintervention, and vaccination acceptance reaching 68.4% [49]. These findings together suggest that theory-driven educational strategies addressing cultural concerns can produce reliably positive effects across Middle East and North Africa populations.

Our observed knowledge gains, rising from 2.97 at baseline to 3.74 at follow-up, mirror results from Chinese college populations who participated in web-based IMB model interventions, where significant increases in HPV knowledge and motivational determinants were consistently reported [40,50,51]. However, a key contrast emerges despite marked improvements in knowledge and intention: Chinese studies reported very limited translation into actual vaccine uptake, a gap attributed to systemic barriers such as high vaccine cost and restricted insurance coverage [52]. This intention-behavior discrepancy highlights that, although educational interventions can effectively enhance cognitive and motivational precursors to vaccination, their population-level impact ultimately depends on structural factors, including affordability, vaccine access, and provider recommendation practices.

Not all educational interventions achieve sustained behavioral impact. Systematic reviews of HPV vaccine education indicate that knowledge gains do not invariably translate into vaccination intentions or uptake, particularly when interventions fail to address context-specific motivational barriers such as perceived stigma, safety concerns, or family opposition [11]. Moreover, intention effects observed at short-term follow-up (1-3 months) often decay by 6 to 12 months absent booster sessions or reminder systems [13]. Our 3-month results should therefore be interpreted as promising but preliminary, requiring longer-term follow-up with objective vaccination outcomes to confirm durability and behavioral translation.

### Theoretical Mechanisms and IMB Model Validation

The intervention’s effectiveness supports IMB model predictions that targeting all 3 constructs, information, motivation, and behavioral skills, produces more robust effects than information-only approaches [38]. The information component addressed widespread misconceptions (HPV/HIV confusion, infertility concerns, and Halal permissibility) and provided evidence-based content on vaccine safety and efficacy, yielding significant knowledge gains. The motivational component emphasized cancer prevention framing rather than STI prevention to reduce stigma, addressed personal susceptibility and severity perceptions, and normalized vaccination within cultural contexts, resulting in improved health awareness and attitudes. The behavioral skills component strengthened self-efficacy, communication competencies for discussing vaccination with providers and family members, and problem-solving for navigating appointment systems, contributing to the substantial intentions effect.

Notably, the progressive increase in intentions from postintervention (4.65) to 3-month follow-up (5.04), rather than decay, suggests that behavior change processes may require extended periods for consolidation as individuals integrate new information, negotiate social contexts, and build decision-making confidence. This temporal pattern aligns with IMB-based intervention studies reporting that behavioral skills acquisition requires longer maturation periods than informational or motivational gains [40].

The moderate-to-large effect sizes observed in this study compare favorably with meta-analytic estimates for health education interventions and exceed typical effects for single-session interventions [33]. Several design features likely contributed to effectiveness: (1) cultural adaptation through systematic formative research addressing local barriers (STI stigma, fertility concerns, and family decision-making dynamics), (2) theoretical grounding ensuring comprehensive coverage of behavioral determinants, (3) female-only facilitation enhancing comfort with sensitive topics, (4) interactive group format promoting social normative influences, and (5) high implementation fidelity (93%) maintaining intervention integrity.

### Implications for Clinical Practice and Health Systems

#### Clinicians

These findings suggest that brief, structured education can be feasibly integrated into primary care encounters to prepare women for informed HPV vaccination discussions. Clinicians should prioritize addressing common misconceptions (fertility effects, HPV/HIV confusion, and Halal status) and emphasize cancer prevention rather than STI prevention to reduce stigma in conservative contexts. Providing culturally adapted written materials (pamphlets and decision aids) at appropriate literacy levels can supplement verbal counseling and facilitate family discussions outside clinical settings.

#### Health Systems

The intervention’s standardized format (45 minutes, 2 facilitators, and 10-15 participants), modest resource requirements, and ability to provide high-fidelity support that scales within existing primary care infrastructure could facilitate integration into routine women’s health services, for example, during well-woman visits, postpartum care, or family planning consultations, and could reach women at teachable moments when preventive health is salient. Group education formats offer additional benefits of peer modeling, normative influence, and cost-efficiency compared with individual counseling.

#### Policy

The findings support integrating HPV vaccination into Saudi Arabia’s national immunization program for adult women, as current recommendations do not include this age group in the publicly funded schedule. This expansion should be paired with broad public education campaigns that address informational and motivational barriers at the population level. Policy planning must also account for persistent structural constraints, most notably vaccine cost and accessibility, given robust evidence that educational interventions alone are insufficient to overcome economic obstacles to uptake, as reflected in studies demonstrating pronounced intention-behavior gaps driven by resource limitations [52]. The Tunisian Ministry of Health’s forthcoming school-based HPV vaccination rollout, anticipated for 2025 and informed by intervention research reporting 68.4% vaccine acceptance among female students [49], provides a regionally relevant policy model. Adaptation to Saudi Arabia, however, requires tailoring to local sociocultural dynamics, including family-centered decision-making processes and the gender-segregated organization of educational environments.

#### Shared Decision-Making

Educational interventions strengthen the informational foundation necessary for SDM by equipping women with knowledge, enhancing motivation, and building confidence to engage in vaccination discussions with providers. However, realizing SDM’s full potential in Saudi health care contexts requires parallel efforts to train clinicians in patient-centered communication, modify directive counseling styles, and create structural supports for family involvement in decisions without undermining women’s autonomous agency [27,28]. Future intervention research should evaluate whether enhanced patient readiness translates into higher-quality SDM processes (eg, measured via the Observing Patient Involvement in Decision Making [OPTION] scale) and whether SDM quality mediates relationships between education and vaccination uptake.

Notably, vaccination coverage is a common indicator of the effectiveness of vaccination strategies. In this context, the study by Guarducci et al [53] provides a valuable example of how an organizational and communication network can be used to implement HPV vaccination coverage for both males and females in the Central Tuscany Health Region. Their pilot model led to a significant increase in coverage, from an average of 49.2% in January 2022 to 63.9% in December 2022 [53]. These results support the idea that coordinated, multifaceted interventions can effectively address HPV vaccination uptake gaps and are consistent with the findings observed in our study. The success of these network-based approaches underscores the importance of integrating organizational strategies and community engagement to improve vaccination coverage.

### Study Strengths

This study has several methodological strengths. First, the intervention was grounded in behavioral theory (IMB model) and developed through systematic cultural adaptation involving formative research with target populations and stakeholders, enhancing both internal validity and local relevance. Second, the study achieved excellent retention (97.7%) across 3 time points, allowing assessment of both immediate and sustained effects. Third, despite nonrandomized allocation, baseline balance across measured covariates (standardized differences <.10) and comprehensive covariate adjustment strengthen confidence in effect estimates. Fourth, high implementation fidelity (93% adherence; substantial interrater reliability κ=0.78) and standardized delivery protocols support replicability. Fifth, the primary care setting and focus on adult women, an underserved population in HPV vaccination research, enhance ecological validity and policy relevance. Finally, comprehensive sensitivity analyses (intention-to-treat, per-protocol, multiple imputation, and alternative covariance structures) confirmed robustness of findings across analytic approaches.

### Study Limitations

Several limitations qualify the interpretation and generalizability of findings.

#### Design Limitations

The nonrandomized, time-based allocation (morning vs afternoon clinic sessions) introduces potential selection bias, as clinic attendance timing may correlate with employment status, caregiving responsibilities, health motivation, and socioeconomic factors. While standardized differences less than 0.10 suggest minimal confounding from measured covariates, unmeasured factors (eg, health consciousness and urgency of care-seeking) could bias effect estimates. Future research should use randomized designs or, when randomization is infeasible, use propensity score matching and instrumental variable approaches to strengthen causal inference. Additionally, participants and facilitators were not blinded to group allocation, introducing potential performance and detection bias. T3 telephone interviewers were only partially blinded and could potentially infer allocation if participants mentioned attending education sessions.

#### Self-Reported Limitations

Outcomes relied exclusively on self-report, introducing potential social desirability bias, particularly for attitudes and intentions. The adapted outcome measures have not been comprehensively validated (eg, confirmatory factor analysis and convergent/discriminant validity) in Saudi populations; pilot testing (n=20) provided only preliminary psychometric support. Differential measurement modes (paper-based at T1/T2; telephone at T3) could introduce mode effects, though sensitivity analyses within the control group suggested minimal bias. Future studies could incorporate validated social desirability scales to statistically control for this bias.

#### Generalizability Limits

The study recruited predominantly educated (65% university degree), urban young women from a single region (Northern Border Region), limiting generalizability to rural populations, older women, those with lower educational attainment, and other Saudi regions with potentially different cultural norms and health care access patterns. Exclusion of pregnant women (a key vaccine-eligible group), health care workers, and those completing the full vaccination series further restricts generalizability. The Northern Border Region’s geographic proximity to Jordan and exposure to cross-border cultural influences may have produced a sample with more liberal attitudes than central or southern Saudi regions.

#### Outcome and Follow-Up Limitations

This study assessed self-reported knowledge, awareness, attitudes, and vaccination intentions rather than verified vaccine initiation or series completion. While intention is a well-established predictor of health behavior, it does not guarantee actual vaccination. Therefore, our findings should be interpreted as preliminary evidence of potential impact, and claims regarding clinical meaningfulness, scalability, policy implementation, and broad applicability should be considered with caution. Future research should incorporate objective measures of vaccination behavior to confirm these findings.

The study measured vaccination intentions, a surrogate end point, rather than verified vaccine uptake. The 3-month follow-up is insufficient to assess vaccine uptake behaviors, which may require extended time frames for appointment scheduling, cost accumulation, and series completion (2-3 doses over 6 months). Unmeasured confounding remains a concern, as the study did not systematically assess male partner or family member attitudes toward HPV vaccination. Male partner and family attitudes likely represent meaningful, unmeasured determinants of HPV vaccination uptake. These factors are likely to exert substantial influence on women’s preventive health behaviors in Saudi Arabia’s collectivist cultural context. In this setting, future research should adopt dyadic or family-level designs evaluating concordance between individual intentions and family support and testing family-centered intervention approaches.

#### Process Evaluation Gaps

The study did not include a formal process evaluation to identify which intervention components were most effective, which women benefited most, or what barriers remained after education. Mediational analyses testing whether knowledge gains mediated attitude and intention improvements would strengthen theoretical understanding. Future studies should incorporate mixed methods process evaluations with qualitative interviews exploring participants’ experiences, decision-making processes, and implementation barriers.

### Future Research Directions

Future research should address the following priorities.

#### Objective Behavioral Outcomes

Conduct trials with 12-month follow-up measuring verified vaccine initiation and series completion through medical record linkage or immunization registry data. Include cost-effectiveness analysis to inform policy decisions about program implementation and scale-up.

#### Multisite Generalizability Studies

Use randomized designs spanning diverse Saudi regions (urban/rural, Northern, Central, and Southern) and demographic strata (age, education, and marital status) to establish intervention effectiveness across heterogeneous populations and identify moderators requiring adaptation.

#### Implementation Research

Conduct hybrid effectiveness-implementation trials evaluating both clinical outcomes and implementation processes (reach, adoption, fidelity, cost, and sustainability) under real-world conditions. Identify barriers and facilitators to routine integration into primary care workflows, training requirements for health care staff, and strategies for maintaining fidelity while allowing contextually appropriate adaptations.

#### Family-Centered Interventions

Develop and test interventions targeting male partners and family decision-makers, given evidence that family attitudes substantially influence women’s vaccination decisions in collectivist contexts. Compare the effectiveness of individual-level, couple-level, and family-level intervention approaches.

#### Structural Barrier Reduction

Evaluate combined interventions addressing both informational/motivational barriers (education) and structural barriers (subsidized vaccines, integrated screening-vaccination services, provider training, and reminder/recall systems). Test whether addressing multiple levels simultaneously yields synergistic effects on uptake.

#### Comparative Effectiveness Research

Compare in-person group education (as tested here) with alternative delivery modalities, including individual counseling, web-based digital interventions, mobile health (mHealth) apps, and hybrid approaches combining digital content with personalized counseling. Identify optimal modality-population matches based on literacy levels, technology access, cultural preferences, and baseline motivation.

### Conclusion

This study provides evidence that a culturally adapted, IMB-based educational intervention delivered in Saudi primary care settings significantly improves HPV-related knowledge, health awareness, attitudes, and vaccination intentions among women aged 18 to 45 years, with medium-to-large effect sizes sustained at 3-month follow-up. Effects were consistent across age, education, and health literacy subgroups, suggesting broad applicability. These findings demonstrate that educational interventions targeting cognitive and motivational barriers are feasible, acceptable, and effective in conservative Middle Eastern contexts where HPV vaccination faces cultural sensitivities.

Nevertheless, these results suggest that educational interventions represent a necessary, though insufficient, component of comprehensive HPV prevention strategies. HPV-related cancers are preventable, yet barriers persist at individual, interpersonal, health care system, and policy levels. Closing the gap between vaccine availability and uptake requires multilevel interventions that simultaneously address knowledge, motivation, structural barriers, and cultural realities. This study represents an important step toward evidence-based, culturally grounded HPV prevention programs that can contribute to cervical cancer reduction and women’s health equity in Saudi Arabia and throughout the Middle Eastern region.

## Appendix

Multimedia Appendix 1 TREND checklist.

Multimedia Appendix 2 Comparison of baseline characteristics: study completers vs noncompleters.

Multimedia Appendix 3 Session scripts, facilitator guides, and educational materials.

Multimedia Appendix 4 Complete subgroup analysis results: effect modification testing.

Multimedia Appendix 5 Assessment of mode effects: telephone vs in-person paper administration.

Multimedia Appendix 6 Model comparison statistics: unadjusted vs adjusted linear mixed-effects models.

## Abbreviations

ASR: age-standardized incidence rate
HPV: human papillomavirus
IARC: International Agency for Research on Cancer
ICO: Information Centre on HPV and Cancer
IMB: information-motivation-behavioral skills
KR-20: Kuder-Richardson formula 20
LOCF: last observation carried forward
mHealth: mobile health
MICE: multivariate imputation by chained equations
MOH: Ministry of Health
OPTION: Observing Patient Involvement in Decision Making
PHC: primary health care
SDM: shared decision-making
SFDA: Saudi Food and Drug Authority
SMOG: Simple Measure of Gobbledygook
STI: sexually transmitted infection
TREND: Transparent Reporting of Evaluations with Nonrandomized Designs
VIF: variance inflation factor

## Data Availability

The datasets used and/or analyzed during the current study are available from the corresponding author on reasonable request.
