# Supplementary material for: Impact of a Culturally Tailored Educational Intervention on Human Papillomavirus Knowledge and Vaccination Intentions Among Women in Saudi Arabia: Quasi-Experimental Study
Source: JMIR Public Health Surveill. 2026 Sep 16;12:e104529. doi: 10.2196/104529 (PMC13628028; doi:10.2196/104529)
Supplement: Supplementary file 1 [file publichealth_v12i1e104529_app1.pdf]

SUPPLEMENTARY S6: TREND CHECKLIST

Transparent Reporting of Evaluations with Nonrandomized Designs

Article Title: Impact of A Culturally Tailored Educational Intervention on HPV Knowledge and Vaccination Intentions Among Women in Saudi Arabia

**TITLE AND ABSTRACT**			
	Title: Indicate the study's design with a commonly used term in the title or the abstract	Title, Abstract	✓ Yes
	Abstract: Structured summary of trial design, methods, results, and conclusions	Abstract	✓ Yes
**INTRODUCTION**			
Background			
	Scientific background and explanation of rationale	Introduction, paragraphs 1-5	✓ Yes
	Specific objectives or hypotheses	Introduction, final paragraph; RQ1-3, H1-3	✓ Yes
**METHODS**			
Participants			
	Eligibility criteria for participants	Methods 2.2, Eligibility criteria	✓ Yes
	Settings and locations where the data were collected	Methods 2.1, 2.2	✓ Yes
	Interventions intended for each study condition and how and when they were actually administered	Methods 2.4, Intervention content and delivery	✓ Yes
Objectives			

	Specific objectives and hypotheses	Introduction, final paragraph	✓ Yes
Outcomes			
	Clearly defined primary and secondary outcome measures	Methods 2.5, Primary outcomes	✓ Yes
	How and when outcome measures were assessed	Methods 2.6, Data collection procedures	✓ Yes
	Any changes to trial outcomes after the trial commenced, with reasons	N/A (no changes)	✓ Yes
Sample Size			
	How sample size was determined and, when applicable, explanation of any interim analyses and stopping rules	Methods 2.3	✓ Yes
Assignment Method			
	Method used to assign participants to study conditions, including details of any restriction (e.g., blocking, stratification, minimization)	Methods 2.2, Allocation procedure	✓ Yes
	Allocation ratio	Methods 2.2 (approximately 1:1)	✓ Yes
Blinding (masking)			
	Whether or not participants, those administering the interventions, and those assessing the outcomes were blinded to study condition assignment; if so, statement regarding how the blinding was accomplished and how it was assessed	Methods 2.6 (partial blinding of T3 interviewers)	✓ Yes
Unit (if applicable)			

	Statistical methods used to compare study groups for primary outcome(s), including complex methods for correlated data	Methods 2.8 (linear mixed models)	✓ Yes
	Statistical methods used for additional analyses, such as subgroup analyses and adjusted analysis	Methods 2.8 (subgroup and sensitivity analyses)	✓ Yes
	Methods for imputing missing data, if used	Methods 2.8 (multiple imputation, LOCF)	✓ Yes
	Statistical software or programs used to conduct analyses	Methods 2.8 (R version 4.3.1)	✓ Yes
**RESULTS**			
Participant flow			
	Flow of participants through each stage of the study: enrollment, assignment, allocation, and intervention exposure, follow-up, analysis (a diagram is strongly recommended)	Results 3.1; Figure 1	✓ Yes
	Enrollment dates defining the periods of recruitment and follow-up	Methods 2.1; Results 3.1 (March-October 2025)	✓ Yes
Recruitment			
	Why the trial ended or was stopped (if applicable)	N/A (completed as planned)	✓ Yes
Baseline Data			
	Baseline demographic and clinical characteristics of participants in each study condition	Results 3.2; Table 1	✓ Yes

	Baseline characteristics for each study condition relevant to specific disease prevention research	Results 3.2; Table 1 (health literacy, vaccination history, family history)	✓ Yes
	Baseline comparisons of those lost to follow-up and those retained, overall and by study condition	Results 3.1; Supplementary Table S1	✓ Yes
	Comparison between study population at baseline and target population of interest	Discussion (generalizability section)	✓ Yes
Numbers analyzed			
	Number of participants (denominator) included in each analysis for each study condition, particularly when the denominators change for different outcomes; statement of the results in absolute numbers when feasible	Results 3.1, 3.3-3.7; all tables	✓ Yes
	Presentation of both relative and absolute measures of effect, if applicable	Results 3.3-3.5; Tables 2-3 (β coefficients, mean differences, effect sizes)	✓ Yes
Outcomes and estimation			
	For each primary and secondary outcome, a summary of results for each estimation study condition, and the estimated effect size and a confidence interval to indicate the precision	Results 3.3-3.5; Tables 2-3	✓ Yes
	Inclusion of null and negative findings	Results 3.6 (null subgroup interactions)	✓ Yes
	Subgroup analyses, including prespecification of when they were done	Results 3.6; Supplementary Table S5	✓ Yes

Ancillary analyses			
	Summary of other analyses performed, including subgroup or restricted analyses, indicating which are pre-specified or exploratory	Results 3.7 (sensitivity analyses)	✓ Yes
Adverse events			
	Summary of all important adverse events or unintended effects in each study condition (for specific guidance see CONSORT for harms)	Methods 2.7; Results (no adverse events)	✓ Yes
**DISCUSSION**			
Interpretation			
	Interpretation of the results, taking into account study hypotheses, sources of potential bias, imprecision of measures, multiplicative analyses, and other limitations or weaknesses of the study	Discussion (all sections)	✓ Yes
	Discussion of results taking into account the mechanism by which the intervention was intended to work (causal pathways) or alternative mechanisms or explanations	Discussion (mechanisms section)	✓ Yes
	Discussion of the success of and barriers to implementing the intervention, fidelity of implementation	Results (93% fidelity); Discussion	✓ Yes
	Discussion of research, programmatic, or policy implications	Discussion (implications section)	✓ Yes
Generalizability			
	Generalizability (external validity) of the trial findings, taking into account the study population, the characteristics of the intervention, length of follow-up, incentives, compliance rates, specific	Discussion (generalizability section)	✓ Yes

	sites/settings involved in the study, and other contextual issues		
	Discussion of barriers to implementability or dissemination	Discussion (implementation considerations)	✓ Yes
	Whether the trial addresses a gap in knowledge and how the trial may lead to change in health care practice or policy	Discussion (significance statement)	✓ Yes
	Sources of funding and other support (such as supply of drugs), role of funders	Funding section (end of manuscript)	✓ Yes
	Ethical review and approval, and consent procedures	Methods 2.1, 2.7	✓ Yes

SUMMARY

Total items: 22
Reported items: 22 (100%)
Overall TREND adherence: Excellent
