# Supplementary material for: Impact of a Culturally Tailored Educational Intervention on Human Papillomavirus Knowledge and Vaccination Intentions Among Women in Saudi Arabia: Quasi-Experimental Study
Source: JMIR Public Health Surveill. 2026 Sep 16;12:e104529. doi: 10.2196/104529 (PMC13628028; doi:10.2196/104529)
Supplement: Supplementary file 2 [file publichealth_v12i1e104529_app2.docx]

**SUPPLEMENTAL TABLE S1**

**Comparison of Baseline Characteristics: Study Completers vs. Non-Completers**

**Purpose:** To assess potential attrition bias by comparing baseline characteristics between participants who completed the 3-month follow-up (n=380) and those lost to follow-up (n=9).

| **Characteristic** | **Completers (n=380)** | **Non-Completers (n=9)** | ***P*-value** | **Effect Size** |
| --- | --- | --- | --- | --- |
| **Demographics** |  |  |  |  |
| Age (years), mean ± SD | 28.6 ± 6.9 | 29.4 ± 7.8 | .712 | *d* = 0.11 |
| Age categories, *n* (%) |  |  | .865 | *V* = 0.04 |
| 18-26 years | 161 (42.4) | 4 (44.4) |  |  |
| 27-35 years | 159 (41.8) | 3 (33.3) |  |  |
| 36-45 years | 60 (15.8) | 2 (22.2) |  |  |
| Female, *n* (%) | 380 (100) | 9 (100) | — | — |
| Marital status, *n* (%) |  |  | .781 | *V* = 0.05 |
| Single | 185 (48.7) | 5 (55.6) |  |  |
| Married | 166 (43.7) | 3 (33.3) |  |  |
| Divorced/Widowed | 29 (7.6) | 1 (11.1) |  |  |
| **Socioeconomic Factors** |  |  |  |  |
| Education level, *n* (%) |  |  | .623 | *V* = 0.07 |
| High school or less | 80 (21.1) | 3 (33.3) |  |  |
| University degree | 247 (65.0) | 5 (55.6) |  |  |
| Postgraduate | 53 (13.9) | 1 (11.1) |  |  |
| Employment status, *n* (%) |  |  | .542 | *V* = 0.06 |
| Employed | 220 (57.9) | 6 (66.7) |  |  |
| Unemployed/Student | 160 (42.1) | 3 (33.3) |  |  |
| Monthly household income (SAR), *n* (%) |  |  | .889 | *V* = 0.03 |
| <10,000 | 101 (26.6) | 2 (22.2) |  |  |
| 10,000-20,000 | 181 (47.6) | 4 (44.4) |  |  |
| >20,000 | 98 (25.8) | 3 (33.3) |  |  |
| **Health-Related Factors** |  |  |  |  |
| Health literacy score (0-50), mean ± SD | 34.0 ± 8.7 | 32.4 ± 9.8 | .596 | *d* = 0.18 |
| Health literacy category, *n* (%) |  |  | .701 | *V* = 0.04 |
| Inadequate/Marginal (<33) | 162 (42.6) | 5 (55.6) |  |  |
| Adequate (≥33) | 218 (57.4) | 4 (44.4) |  |  |
| Previous vaccination experience, *n* (%) | 280 (73.7) | 7 (77.8) | .788 | *V* = 0.03 |
| HPV vaccination history (partial), *n* (%) | 14 (3.7) | 0 (0) | .599 | *V* = 0.09 |
| Family history of cervical cancer, *n* (%) | 39 (10.3) | 1 (11.1) | .936 | *V* = 0.01 |
| Regular gynecological check-ups, *n* (%) | 170 (44.7) | 4 (44.4) | .986 | *V* = 0.00 |
| **Baseline Outcome Measures** |  |  |  |  |
| Knowledge score (1-7), mean ± SD | 2.96 ± 1.41 | 2.78 ± 1.52 | .691 | *d* = 0.12 |
| Health awareness score (1-7), mean ± SD | 2.99 ± 1.42 | 3.12 ± 1.68 | .781 | *d* = 0.09 |
| Attitudes score (1-7), mean ± SD | 2.92 ± 1.41 | 2.89 ± 1.55 | .948 | *d* = 0.02 |
| Vaccination intentions (1-7), mean ± SD | 3.99 ± 1.06 | 3.82 ± 1.14 | .619 | *d* = 0.16 |
| **Study Group Allocation** |  |  |  |  |
| Intervention group, *n* (%) | 190 (50.0) | 4 (44.4) | .750 | *V* = 0.03 |
| Control group, *n* (%) | 190 (50.0) | 5 (55.6) |  |  |
| **Reason for Non-Completion** |  |  |  |  |
| Withdrew before intervention/baseline | — | 6 (66.7) | — | — |
| Lost to follow-up after baseline | — | 3 (33.3) | — | — |

**Notes:**

1. **Statistical tests:**
   - Continuous variables: Independent-samples *t*-tests (or Mann-Whitney U if non-normal)
   - Categorical variables: χ² tests (or Fisher's exact test if expected cell count <5)
2. **Effect sizes:**
   - *d* = Cohen's *d* for continuous variables (small: 0.20, moderate: 0.50, large: 0.80)
   - *V* = Cramér's *V* for categorical variables (small: 0.10, moderate: 0.30, large: 0.50)
3. **Interpretation:**
   - All *p*-values > 0.40, indicating no statistically significant differences between completers and non-completers
   - All effect sizes small (<0.20), indicating minimal practical differences
   - Balanced loss across study groups (4 intervention vs. 5 control, ***P*** = 0.750)
4. **Conclusion:** The absence of significant baseline differences between completers and non-completers, combined with the very low attrition rate (2.3%), suggests **minimal attrition bias**. Missing data appears to be **missing completely at random (MCAR)**, supporting the validity of complete-case analysis as the primary approach.
5. **Reasons for non-completion:**
   - 6 participants withdrew before receiving assigned condition (2 intervention pre-session, 4 control pre-assessment)
   - 3 participants lost to follow-up after baseline (2 intervention, 1 control)
   - Reasons: 2 relocation, 1 lost contact, 6 scheduling conflicts/withdrawal of consent

**Abbreviations:** SD: Standard Deviation; SAR: Saudi Riyal; *d*: Cohen's *d* effect size; *V*: Cramér's *V* effect size; MCAR: Missing Completely At Random.

**Analysis software:** R version 4.3.1 (R Core Team, 2023)
