# Supplementary material for: Impact of a Culturally Tailored Educational Intervention on Human Papillomavirus Knowledge and Vaccination Intentions Among Women in Saudi Arabia: Quasi-Experimental Study
Source: JMIR Public Health Surveill. 2026 Sep 16;12:e104529. doi: 10.2196/104529 (PMC13628028; doi:10.2196/104529)
Supplement: Supplementary file 3 [file publichealth_v12i1e104529_app3.docx]

**SUPPLEMENTARY S2**

**Intervention Protocol: Structured Educational Session on HPV Vaccination for Young Women in Saudi Arabia**

**1. INTRODUCTION**

**1.1 Background and Rationale**

Human papillomavirus (HPV) represents a significant public health challenge in Saudi Arabia, with persistent high-risk HPV infection serving as the primary etiological agent for cervical cancer. Despite the proven efficacy and safety of prophylactic HPV vaccines, uptake among age-eligible Saudi women remains critically low, estimated at approximately 10.9% for series initiation. This suboptimal coverage stems from multiple barriers including limited disease awareness, vaccine misconceptions, cultural and religious sensitivities, and inadequate healthcare provider education on shared clinical decision-making (SCDM) approaches to vaccination counseling.

Educational interventions grounded in behavioral theory have demonstrated effectiveness in improving vaccination knowledge, attitudes, and intentions across diverse populations. However, few such interventions have been culturally adapted for conservative Middle Eastern contexts, and none have been rigorously evaluated in Saudi Arabia's Northern Border Region—a geographically isolated area characterized by limited access to preventive health services, traditional social norms, and minimal prior research on HPV vaccination attitudes.

This intervention protocol describes a theory-driven, culturally adapted group educational session designed to address the identified knowledge gaps, motivational barriers, and behavioral skills deficits that impede informed decision-making about HPV vaccination among women aged 18–45 years in the Northern Border Region. The intervention integrates evidence-based health communication strategies with culturally congruent messaging to facilitate authentic shared clinical decision-making while respecting local values and religious beliefs.

**1.2 Theoretical Framework:** The Information-Motivation-Behavioral Skills (IMB) Model

The intervention is grounded in the Information-Motivation-Behavioral Skills (IMB) model, a well-established framework for understanding and promoting health behavior change. The IMB model posits that health behavior is a function of three core determinants:

**1. Information: Accurate, relevant knowledge about the health issue and preventive behavior. For HPV vaccination, this includes understanding:**

- The nature of HPV infection and its relationship to cervical cancer
- Vaccine mechanism of action, efficacy, and safety profile
- Recommended vaccination schedules and access pathways
- Benefits and risks of vaccination

**2. Motivation: Personal and social incentives to engage in the health behavior, encompassing:**

- *Personal motivation:* Perceived personal vulnerability to HPV and cervical cancer, anticipated benefits of vaccination, and positive attitudes toward vaccines
- *Social motivation:* Perceived social norms regarding vaccination, family and community support, and endorsements from trusted sources (healthcare providers, religious leaders)

**3. Behavioral Skills: The objective abilities and perceived self-efficacy to perform the health behavior, including:**

- *Objective skills:* Practical competencies such as making vaccination appointments, communicating with healthcare providers, and navigating the healthcare system
- *Perceived self-efficacy:* Confidence in one's ability to overcome barriers and successfully complete vaccination

According to the IMB model, these three components work synergistically: Information and Motivation directly influence Behavioral Skills, which in turn directly affect health behavior (in this case, HPV vaccination uptake). Information and Motivation may also exert direct effects on behavior when the required behavioral skills are minimal or already well-established.

**Application to HPV Vaccination in Saudi Arabia:**

**The IMB model was selected for this intervention based on:**

1. Empirical Support: Extensive evidence demonstrates the model's effectiveness in predicting and promoting preventive health behaviors, including vaccination, across diverse cultural contexts.
2. Comprehensive Coverage: The model addresses the multifaceted nature of vaccination decisions, encompassing cognitive (knowledge), affective (attitudes), social (norms), and behavioral (skills) dimensions.
3. Cultural Adaptability: The model's flexibility allows for culturally specific content within each component, enabling tailoring to conservative Saudi Arabian contexts without compromising theoretical integrity.
4. Shared Decision-Making Alignment: By emphasizing both information provision and skills development, the model naturally supports SCDM processes wherein patients and providers collaborate to make values-concordant healthcare decisions.

**The intervention operationalizes each IMB component through specific session modules:**

- Information Component: Modules 1 and 2 provide foundational HPV knowledge and vaccine information
- Motivation Component: Modules 2, 3, and 4 address personal risk perception, social norms, and attitudinal barriers
- Behavioral Skills Component: Module 3 and the Action Planning segment build communication skills, decision-making competencies, and self-efficacy

**1.3 Cultural Adaptation Process**

Recognizing that direct translation of Western health interventions often fails in non-Western contexts due to cultural incongruence, we employed a rigorous four-phase cultural adaptation process:

**Phase 1: Information Gathering :**

- Conducted 20 semi-structured interviews with community stakeholders, including women aged 18–45, male family decision-makers, primary care physicians, religious scholars (*ulama*), and community health workers
- Explored existing HPV knowledge, vaccination attitudes, cultural values, religious beliefs, preferred information sources, and perceived barriers
- Identified culturally appropriate terminology (e.g., framing HPV as a "health threat" rather than "sexually transmitted infection" to reduce stigma)
- Documented preferred educational formats and facilitator characteristics

**Key Findings:**

- Strong preference for gender-concordant (female) facilitators
- High value placed on religious authority endorsements
- Concerns about vaccine impact on fertility and cultural perceptions of promiscuity
- Limited awareness of cervical cancer as a preventable disease
- Desire for family-inclusive decision-making approaches

**Phase 2: Preliminary Adaptation**

- Multidisciplinary team (health educators, cultural advisors, religious scholars, gynecologists, public health experts) reviewed formative findings
- Developed culturally adapted intervention content emphasizing:
  - Cancer prevention framing rather than STI prevention to minimize stigma
  - Family health benefits including protection of future children
  - Religious permissibility with explicit endorsements from Islamic scholars
  - Modesty and privacy through female-only sessions and female facilitators
  - Collective decision-making acknowledging family and spousal involvement
- Created Arabic-language materials using Modern Standard Arabic (accessible across dialects) with sixth-grade reading level
- Developed visual aids featuring local imagery and culturally appropriate depictions
- Integrated testimonials from respected Saudi women who chose vaccination

**Phase 3: Pilot Testing**

- Conducted three pilot sessions with 20 women not included in the main study
- Assessed content comprehension, cultural acceptability, session duration, and engagement
- Gathered qualitative feedback through post-session focus groups

**Pilot Feedback:**

- High acceptability ratings (mean 4.6/5.0)
- Requests for more specific information on vaccine access and cost
- Suggestions to include more visual demonstrations of vaccine administration
- Positive reception of testimonial videos and religious endorsements

**Phase 4: Refinement**

- Revised intervention materials based on pilot feedback
- Enhanced Module 2 with detailed access pathway information and cost transparency
- Added brief video demonstrating vaccine injection procedure to demystify process
- Strengthened action planning segment with specific local clinic contact information
- Finalized facilitator training manual with cultural sensitivity guidelines

This iterative adaptation process ensured that the intervention maintained theoretical fidelity to the IMB model while achieving cultural congruence with Northern Border Region norms and values.

**1.4 Intervention Objectives**

The structured educational session aims to achieve the following specific, measurable objectives:

**Primary Objectives:**

1. **Increase HPV and Cervical Cancer Knowledge**
   - Participants will correctly identify HPV as the primary cause of cervical cancer
   - Participants will recognize high-risk HPV types and transmission routes
   - Participants will understand the natural history of HPV infection
   - Participants will articulate the relationship between persistent HPV infection and cervical cancer development
2. **Enhance HPV Vaccine Knowledge**
   - Participants will describe the mechanism of action and types of HPV vaccines
   - Participants will accurately report vaccine efficacy rates and safety profiles
   - Participants will identify recommended vaccination schedules and eligible age groups
   - Participants will recognize access pathways and cost considerations in Saudi Arabia
3. **Improve Vaccination Attitudes**
   - Participants will perceive HPV vaccination as beneficial for personal health
   - Participants will view vaccines as safe and appropriate within cultural/religious contexts
   - Participants will express reduced concerns about fertility impacts and social stigma
   - Participants will demonstrate positive affect toward vaccination consideration
4. **Strengthen Vaccination Intentions**
   - Participants will express increased likelihood of accepting HPV vaccination
   - Participants will indicate willingness to discuss vaccination with healthcare providers
   - Participants will report intentions to share information with family members
   - Participants will demonstrate commitment to taking actionable next steps

**Secondary Objectives:**

1. **Build Self-Efficacy for Shared Decision-Making**
   - Participants will articulate their personal values regarding vaccination
   - Participants will prepare questions to ask healthcare providers
   - Participants will demonstrate confidence in navigating the healthcare system
   - Participants will express ability to engage in informed decision-making discussions
2. **Develop Action Planning Skills**
   - Participants will create personalized vaccination action plans
   - Participants will identify potential barriers and mitigation strategies
   - Participants will specify timelines for vaccination-related activities
   - Participants will document concrete next steps (e.g., clinic appointments)
3. **Address Misconceptions and Reduce Stigma**
   - Participants will refute common myths about HPV vaccines using evidence
   - Participants will recognize HPV as a common, medically manageable infection
   - Participants will demonstrate reduced shame and stigma associated with HPV discussion
   - Participants will express willingness to discuss HPV openly with peers

**1.5 Target Population**

**Inclusion Criteria:**

- Saudi women aged 18–45 years (age range consistent with extended HPV vaccination recommendations and reproductive health considerations)
- Residents of Northern Border Region communities
- Arabic-speaking (able to understand Modern Standard Arabic)
- Not currently pregnant (as pregnancy is a temporary contraindication for HPV vaccination)
- Able to provide informed consent

**Exclusion Criteria:**

- Previous HPV vaccination (three-dose series completion)
- Current pregnancy (confirmed or self-reported)
- Cognitive impairment precluding informed consent or comprehension
- Severe psychiatric illness requiring active treatment
- Non-Arabic speakers or those unable to comprehend intervention content

**Rationale for Age Range: The 18–45 age range was selected based on:**

- Saudi Arabian age of consent and autonomous medical decision-making (≥18 years)
- US Advisory Committee on Immunization Practices (ACIP) recommendations for routine vaccination through age 26 and shared clinical decision-making for ages 27–45
- Reproductive health relevance (peak childbearing years in Saudi context)
- Feasibility of cervical cancer prevention (prior to likely HPV exposure consequences)

**1.6 Intervention Setting and Format**

**Setting:**

- Primary healthcare centers in Northern Border Region, Saudi Arabia
- Private conference rooms or health education spaces within clinics
- Comfortable seating arranged in circular or U-shaped configuration to facilitate discussion and interaction
- Quiet environment free from clinical interruptions

**Format:**

- Group-based delivery: Small groups of 10–15 participants to enable personalized attention while leveraging peer learning and social support
- Single-session design: 45-minute structured session balancing comprehensiveness with participant time constraints
- Interactive pedagogy: Combination of didactic presentation, facilitated discussion, role-play activities, and multimedia content
- Gender-concordant facilitation: Two female health educators per session to ensure cultural appropriateness and participant comfort

**Rationale for Group Format: Group-based delivery was selected over individual counseling based on:**

- Efficiency: Ability to reach more participants with limited healthcare educator resources
- Social learning: Peer modeling and shared experiences enhance engagement and normalize vaccination consideration
- Cost-effectiveness: Reduced per-participant delivery costs support scalability
- Cultural appropriateness: Women-only groups align with gender segregation norms in conservative Saudi contexts
- Empirical support: Evidence suggests group health education can be as effective as individual counseling for knowledge and attitude change

**1.7 Facilitator Qualifications and Training**

**Facilitator Qualifications:**

- Female health educators with minimum of diploma-level training in nursing, health education, or related fields
- Fluency in Modern Standard Arabic and local dialects
- Prior experience in health education or patient counseling (preferred)
- Cultural competence and sensitivity to Northern Border Region norms

**Training Program (2-Day Intensive):**

***Day 1: Content Knowledge and Educational Principles***

- Hour 1–2: HPV and cervical cancer epidemiology, pathophysiology, and prevention
- Hour 3–4: HPV vaccine types, mechanisms, efficacy, safety, and access in Saudi Arabia
- Hour 5–6: IMB model overview and application to vaccination behavior
- Hour 7–8: Adult learning principles, facilitation techniques, and group management strategies

***Day 2: Cultural Competence and Session Delivery***

- Hour 1–2: Cultural and religious considerations in Northern Border Region; appropriate terminology and framing
- Hour 3–4: Shared decision-making principles and motivational interviewing techniques
- Hour 5–6: Session protocol review, module-by-module walkthrough, and material familiarization
- Hour 7–8: Practice facilitation with feedback, fidelity monitoring procedures, and ethical considerations

**Ongoing Support:**

- Supervision by senior health educators during initial sessions
- Bi-weekly facilitator debriefing meetings to address challenges and share strategies
- Access to resource library with FAQs, scientific references, and troubleshooting guides

**1.8 Intervention Fidelity**

Intervention fidelity—the degree to which the intervention is delivered as intended—is critical for internal validity and replicability. We employed multiple strategies to ensure high fidelity:

**Fidelity Monitoring Plan:**

1. **Standardized Protocol:**
   - Detailed facilitator manual with scripted content, timing guidelines, and activity instructions
   - Structured session outline ensuring consistent content delivery across groups and facilitators
2. **Fidelity Checklist:**
   - 15-item checklist assessing adherence to protocol elements (e.g., "Did facilitator cover HPV transmission routes?", "Did facilitator address fertility misconceptions?")
   - Binary scoring (1=completed, 0=not completed) with fidelity percentage calculated as (items completed ÷ 15) × 100%
3. **Audio Recording:**
   - Random selection of 20% of sessions for audio recording (with participant consent)
   - Independent review by two trained raters not involved in session delivery
   - Inter-rater reliability assessed using Cohen's kappa (target ≥0.80)
4. **Real-Time Observation:**
   - Supervisory observation of 10% of sessions using structured observation forms
   - Immediate post-session feedback to facilitators on protocol adherence and areas for improvement
5. **Facilitator Self-Report:**
   - Post-session reflection logs documenting deviations from protocol, participant questions, and implementation challenges
   - Aggregate review to identify systematic implementation issues

**Fidelity Threshold:**

- Target fidelity: ≥90% protocol adherence across monitored sessions
- Achieved fidelity: Mean 93% (range 89–97%), indicating excellent implementation consistency

**Adaptations Allowed:**

- Flexible response to spontaneous participant questions (content must remain evidence-based)
- Minor timing adjustments to accommodate group pace (±3 minutes per module)
- Examples and analogies adapted to participants' local contexts and experiences
- All adaptations documented in facilitator logs for transparency

**1.9 Ethical Considerations**

**Informed Consent:**

- All participants provided written informed consent prior to participation
- Consent forms detailed study purpose, procedures, risks/benefits, confidentiality protections, and right to withdraw
- Participants informed that study participation would not affect their clinical care

**Autonomy and Decision-Making:**

- Intervention explicitly framed as supporting *informed decision-making*, not persuading or coercing vaccination
- Content presented benefits and risks objectively, acknowledging vaccination as a personal choice
- Respect for participants' cultural values, religious beliefs, and family decision-making processes

**Privacy and Confidentiality:**

- All sessions conducted in private rooms with no non-participants present (except approved family members if requested)
- Participants assigned unique study identifiers; personal identifiers stored separately from data
- Audio recordings stored on encrypted, password-protected devices accessible only to authorized research staff
- Data retention: Five years post-study completion, then securely destroyed

**Minimizing Harm:**

- Careful attention to culturally sensitive framing to avoid stigma or shame
- Facilitators trained to recognize and respond to participant distress
- Immediate referral protocols for participants expressing psychological distress or urgent health concerns

**Post-Study Education for Control Group:**

- To ensure ethical equipoise, control group participants received full educational materials upon completion of 3-month follow-up
- Access provided to online educational video and vaccination clinic contact information

**Ethical Approval:**

- Bioethical Research Committee of Northern Border University (approval NBU-HAP-09-A-043)
- Study conducted in accordance with Declaration of Helsinki and Saudi Arabian research ethics guidelines

**1.10 Supplementary Materials Overview**

This supplementary document provides comprehensive details on the intervention protocol to enable replication and adaptation by other researchers and practitioners. The following sections include:

- Section 2: Detailed session schedule and module content
- Section 3: Facilitator training curriculum
- Section 4: Fidelity monitoring tools and procedures
- Section 5: Participant materials (pamphlet, handouts, visual aids)
- Section 6: Evaluation questionnaires and scoring guidelines
- Section 7: Implementation considerations and lessons learned

**1.11 Intended Audience**

**This supplementary material is designed for:**

- Researchers seeking to replicate or adapt the intervention for different populations or settings
- Healthcare Administrators evaluating feasibility of implementing structured HPV vaccination education in primary care
- Public Health Practitioners developing vaccination promotion programs in conservative or low-resource contexts
- Health Educators requiring detailed training materials and session protocols
- Policy Makers assessing evidence-based approaches to increasing HPV vaccination coverage in Saudi Arabia

**1.12 Acknowledgments**

We gratefully acknowledge the contributions of community stakeholders, religious scholars, healthcare providers, and pilot study participants whose insights shaped this culturally adapted intervention. We also thank the facilitators who delivered the intervention with dedication and cultural sensitivity, and the research assistants who ensured rigorous data collection and fidelity monitoring.

**INTERVENTION SCHEDULE TABLES**

**Table 1. Intervention Group Session Schedule and Content Delivery Protocol**

| **Session Component** | **Duration (minutes)** | **Delivery Method** | **Content Overview** | **Materials/Activities** | **Learning Objectives** |
| --- | --- | --- | --- | --- | --- |
| **Pre-Session Preparation** | 10 | - | Registration and consent verification | - Attendance register - Name tags - Pre-session questionnaire | - Confirm participation - Establish rapport - Assess baseline readiness |
| **Welcome and Introduction** | 5 | Interactive lecture | - Welcome remarks - Session overview - Ground rules - Icebreaker activity | - PowerPoint slides - Visual aids - Participant introductions | - Create safe learning environment - Establish group cohesion - Clarify session expectations |
| **Module 1: Understanding HPV and Cervical Cancer** | 10 | Didactic presentation with visual aids | - HPV definition and prevalence - Transmission routes - High-risk vs. low-risk HPV types - Natural history of infection - Cervical cancer development - Disease burden in Saudi Arabia | - Anatomical diagrams - Epidemiological infographics - Local prevalence data charts - Case vignettes | - Define HPV and identify transmission modes - Differentiate HPV types - Explain HPV-cervical cancer link - Recognize local disease burden |
| **Module 2: HPV Vaccines—Safety, Efficacy, and Access** | 12 | Interactive presentation with Q&A | - Vaccine types and mechanisms - Efficacy data (clinical trials) - Safety profile and adverse events - Contraindications - Vaccine availability in Saudi Arabia - Cost and insurance coverage - Administration schedule | - Vaccine information sheets - Safety data graphics - FAQs handout - Access pathway flowchart - Short testimonial video (2 min) | - Describe vaccine mechanism and types - Recognize vaccine efficacy and safety - Identify access points for vaccination - Understand administration regimen |
| **Module 3: Making Informed Decisions About Vaccination** | 13 | Facilitated discussion and role-play | - Shared decision-making principles - Benefits vs. risks assessment - Personal values clarification - Communication with healthcare providers - Family and partner discussions - Religious and cultural considerations | - Decision aid tool - Role-play scenarios - Provider-patient dialogue scripts - Values clarification worksheet - Religious leader endorsement quotes | - Apply shared decision-making framework - Assess personal benefits/risks - Prepare questions for providers - Navigate cultural/religious contexts - Engage family in decision process |
| **Module 4: Addressing Common Misconceptions** | 10 | Interactive myth-busting activity | - Myth: Vaccines cause infertility - Myth: Only sexually active need vaccine - Myth: Vaccine promotes promiscuity - Myth: HPV only affects women - Myth: Natural immunity is sufficient - Addressing stigma and shame | - Myth vs. Fact cards - Evidence-based fact sheets - Group discussion prompts - Peer testimonials - Cultural sensitivity examples | - Identify and refute common myths - Use evidence to counter misinformation - Reduce stigma and shame - Build confidence in vaccine information |
| **Summary and Action Planning** | 5 | Facilitated goal-setting | - Key message recap - Personal action plan development - Next steps identification - Setting vaccination appointment intentions - Questions and concerns | - Action plan template - Appointment reminder cards - Resource contact list - Summary handout | - Synthesize key learnings - Develop personal vaccination plan - Identify barriers and solutions - Commit to next steps |
| **Evaluation and Closure** | 5 | Self-administered assessment | - Immediate post-session questionnaire - Session feedback form - Distribution of take-home materials - Contact information for follow-up questions | - Post-session questionnaire - Feedback forms - 8-page Arabic pamphlet - Facilitator contact cards | - Assess immediate knowledge gain - Gather session feedback - Provide ongoing support resources |
| **Total Session Duration** | **45** | - | - | - | - |

**Session Logistics:**

- **Group Size:** 10–15 participants per session
- **Facilitators:** Two female certified health educators
- **Language:** Modern Standard Arabic
- **Setting:** Private conference room in primary healthcare center
- **Seating:** Circular or U-shaped arrangement to facilitate discussion
- **Equipment:** Projector, laptop, flip chart, audio system
- **Refreshments:** Light snacks and beverages provided

**Facilitator Training:**

- 2-day training program covering:
  - HPV and cervical cancer content knowledge
  - Adult learning principles and facilitation techniques
  - Cultural sensitivity and religious considerations
  - Motivational interviewing skills
  - Session protocol and fidelity monitoring

**Fidelity Monitoring:**

- 20% of sessions audio-recorded
- Independent raters assess adherence using standardized checklist
- Mean fidelity target: ≥90% protocol adherence

**Table 2. Control Group Standard Care Schedule and Content Exposure**

| **Timepoint** | **Duration (minutes)** | **Activity** | **Content Provided** | **Personnel Involved** | **Location** | **Materials** |
| --- | --- | --- | --- | --- | --- | --- |
| **Baseline (Time 1)** | 20–30 | Standard clinic visit | - Routine health assessment - General preventive health counseling - No specific HPV education - Baseline questionnaire administration | - Primary care physician - Clinic nurse - Research assistant | Primary healthcare center consultation room | - Standard clinic forms - Baseline questionnaire - Routine health pamphlets |
| **Post-Baseline (Week 1)** | 15–20 | Standard follow-up | - Brief health check - General wellness advice - No structured HPV intervention - Time 2 questionnaire administration | - Clinic nurse - Research assistant | Primary healthcare center | - Time 2 questionnaire - Standard health materials |
| **3-Month Follow-Up (Time 3)** | 15–20 | Telephone interview | - General health status inquiry - Assessment of any health changes - Time 3 questionnaire administration - No additional HPV education | - Trained female interviewer (blinded to study purpose) | Telephone (remote) | - Standardized telephone script - Time 3 questionnaire |

**Standard Care Description:**

**Routine Primary Care Services (Control Group):**

Control group participants received standard clinical care available at all participating primary healthcare centers in the Northern Border Region, consisting of:

1. **Initial Consultation (Baseline Visit):**
   - General physical examination by primary care physician
   - Routine health history documentation
   - Standard preventive health counseling (diet, exercise, chronic disease management)
   - No targeted HPV or cervical cancer education beyond routine gynecological health mentions
   - Duration: 20–30 minutes
2. **Standard Health Information Exposure:**
   - Access to general health posters and pamphlets in clinic waiting areas
   - Generic women's health brochures (may include brief cervical cancer mention)
   - No structured educational sessions or group interventions
   - No dedicated HPV vaccination counseling
3. **Physician-Patient Interaction:**
   - Standard medical consultation format
   - HPV or vaccination mentioned only if patient initiated inquiry
   - No systematic use of decision aids or educational tools
   - No formalized shared decision-making protocol for HPV vaccination
4. **Follow-Up Procedures:**
   - Time 2 assessment approximately 1 week post-baseline (mean 6.8 days, SD=1.2)
   - Conducted by clinic nurse or research assistant in clinic setting
   - Questionnaire administration only; no additional educational content
   - Duration: 15–20 minutes
5. **3-Month Telephone Assessment:**
   - Standardized telephone interview by trained blinded assessor
   - Assessment of health status and outcome measures
   - No educational intervention or health coaching
   - Duration: 15–20 minutes

**Control Group Ethical Considerations:**

To ensure ethical equipoise and minimize potential harm from knowledge withholding:

- **Post-Study Education:** Upon completion of 3-month follow-up, all control group participants received:
  - The same 8-page Arabic HPV vaccination pamphlet provided to intervention group
  - Access to online educational video (15-minute condensed version of intervention content)
  - Contact information for vaccination clinics and counseling services
- **Immediate Referral Protocol:** Any control participant expressing urgent health concerns or requesting HPV information during study period was:
  - Immediately referred to appropriate healthcare services
  - Provided basic factual information without compromising study integrity
  - Documented as protocol deviation with reasons
- **Monitoring:** Control group participants were monitored for adverse events or significant health changes at each assessment timepoint

**Table 3. Comparative Timeline of Intervention vs. Control Group Activities**

| **Study Phase** | **Time Period** | **Intervention Group Activities** | **Control Group Activities** | **Both Groups** |
| --- | --- | --- | --- | --- |
| **Recruitment** | November–December 2025 | Approached in clinic waiting areas Eligibility screening Informed consent | Approached in clinic waiting areas Eligibility screening Informed consent | - Baseline questionnaire (Time 1) - Demographics collection - Health literacy assessment |
| **Intervention Delivery** | January–February 2025 | 45-minute structured group educational session (n=194 participants, ~13 sessions) Fidelity monitoring | Routine standard care only No structured HPV education | - |
| **Immediate Post-Intervention** | January–February 2025 (~1-week post-baseline) | Time 2 questionnaire administration Assessment in clinic | Time 2 questionnaire administration Assessment in clinic | - Immediate knowledge assessment - Attitudes and intentions measurement |
| **Follow-Up Assessment** | March–May 2025 (3 months post-baseline) | Telephone interview by blinded assessor Time 3 questionnaire | Telephone interview by blinded assessor Time 3 questionnaire | - Knowledge retention - Attitudes and intentions - Self-reported vaccination uptake |
| **Post-Study Education** |  | - | Educational materials provided: - 8-page pamphlet - Online video access - Vaccination clinic referrals | Study completion certificates Compensation (if applicable) |

**Table 4. Intervention Content Mapping to IMB Model Components**

| **IMB Component** | **Session Modules Addressing Component** | **Specific Content Elements** | **Expected Behavioral Outcome** |
| --- | --- | --- | --- |
| **Information** | Module 1: Understanding HPV and Cervical Cancer Module 2: Vaccines—Safety, Efficacy, Access | - HPV transmission and types - Disease natural history - Cervical cancer risk - Vaccine mechanism - Efficacy and safety data - Local epidemiology | **Increased HPV knowledge** **Enhanced health awareness** |
| **Motivation** | Module 2: Vaccines—Safety, Efficacy, Access Module 3: Making Informed Decisions Module 4: Addressing Misconceptions | - Personal risk assessment - Benefits perception - Social norms and peer testimonials - Religious/cultural endorsements - Myth refutation - Values clarification | **Positive vaccination attitudes** **Stronger intentions to vaccinate** |
| **Behavioral Skills** | Module 3: Making Informed Decisions Summary and Action Planning | - Provider communication skills - Shared decision-making practice - Appointment scheduling - Family discussion strategies - Action planning - Barrier identification and problem-solving | **Increased self-efficacy** **Vaccination behavior initiation** |

**NOTES:**

1. **Session Frequency:** Intervention sessions conducted 3–4 times weekly to accommodate group sizes of 10–15 participants
2. **Makeup Sessions:** Participants unable to attend scheduled session offered alternative time within same week
3. **Attendance:** Minimum 80% session attendance (≥36 minutes) required for per-protocol analysis
4. **Documentation:** All sessions documented with attendance logs, fidelity checklists, and facilitator field notes
5. **Cultural Adaptations:** All materials reviewed by cultural advisors and religious scholars for appropriateness
6. **Language:** All verbal and written content delivered in Modern Standard Arabic; materials at 6th-grade reading level
