# Supplementary material for: Impact of a Culturally Tailored Educational Intervention on Human Papillomavirus Knowledge and Vaccination Intentions Among Women in Saudi Arabia: Quasi-Experimental Study
Source: JMIR Public Health Surveill. 2026 Sep 16;12:e104529. doi: 10.2196/104529 (PMC13628028; doi:10.2196/104529)
Supplement: Supplementary file 4 [file publichealth_v12i1e104529_app4.docx]

**SUPPLEMENTARY S3**

**Complete Subgroup Analysis Results: Effect Modification Testing**

**Purpose:** To evaluate whether intervention effects varied across prespecified demographic and health-related subgroups using three-way interactions (Time × Group × Moderator).

**Statistical approach:** Bonferroni-corrected α = 0.0125 per outcome domain (testing 4 moderators separately within each outcome).

**Table S5A. KNOWLEDGE SCORE (1-7 Scale): Subgroup Results**

**Stratified 3-Month Change Scores (T3 - T1)**

| **Moderator** | **Subgroup** | **n** | **Intervention** | **Control** | **Between-Group** |
| --- | --- | --- | --- | --- | --- |
|  |  |  | **Mean Δ (95% CI)** | **Mean Δ (95% CI)** | **Difference (95% CI)** |
| **Age** | 18-26 years | 161 | 0.82 (0.56, 1.08) | -0.09 (-0.35, 0.17) | 0.91 (0.54, 1.28) |
|  | 27-45 years | 219 | 0.74 (0.52, 0.96) | -0.05 (-0.27, 0.17) | 0.79 (0.48, 1.10) |
| **Education** | University | 247 | 0.85 (0.61, 1.09) | -0.03 (-0.27, 0.21) | 0.88 (0.54, 1.22) |
|  | <University | 133 | 0.63 (0.32, 0.94) | -0.13 (-0.44, 0.18) | 0.76 (0.32, 1.20) |
| **Health Literacy** | Adequate (≥33) | 218 | 0.89 (0.64, 1.14) | 0.01 (-0.24, 0.26) | 0.88 (0.52, 1.24) |
|  | Inadequate (<33) | 162 | 0.61 (0.32, 0.90) | -0.18 (-0.47, 0.11) | 0.79 (0.38, 1.20) |
| **Prior Vaccination** | Any experience | 280 | 0.81 (0.58, 1.04) | -0.04 (-0.27, 0.19) | 0.85 (0.52, 1.18) |
|  | No experience | 100 | 0.69 (0.33, 1.05) | -0.14 (-0.50, 0.22) | 0.83 (0.33, 1.33) |

**Three-Way Interaction Tests**

| **Moderator** | **F-statistic** | ***P-*value** | **Significant?** |
| --- | --- | --- | --- |
| Time × Group × Age | 0.89 | .412 | No |
| Time × Group × Education | 0.64 | .529 | No |
| Time × Group × Health Literacy | 0.52 | .596 | No |
| Time × Group × Prior Vaccination | 0.18 | .835 | No |

**Conclusion:** No significant effect modification for knowledge. Intervention effective across all subgroups.

**Table S5B. HEALTH AWARENESS SCORE (1-7 Scale): Subgroup Results**

**Stratified 3-Month Change Scores (T3 - T1)**

| **Moderator** | **Subgroup** | **n** | **Intervention** | **Control** | **Between-Group** |
| --- | --- | --- | --- | --- | --- |
|  |  |  | **Mean Δ (95% CI)** | **Mean Δ (95% CI)** | **Difference (95% CI)** |
| **Age** | 18-26 years | 161 | 0.95 (0.67, 1.23) | 0.10 (-0.18, 0.38) | 0.85 (0.45, 1.25) |
|  | 27-45 years | 219 | 0.82 (0.58, 1.06) | 0.06 (-0.18, 0.30) | 0.76 (0.43, 1.09) |
| **Education** | University | 247 | 0.96 (0.70, 1.22) | 0.11 (-0.15, 0.37) | 0.85 (0.48, 1.22) |
|  | <University | 133 | 0.72 (0.39, 1.05) | 0.03 (-0.30, 0.36) | 0.69 (0.22, 1.16) |
| **Health Literacy** | Adequate (≥33) | 218 | 0.98 (0.71, 1.25) | 0.14 (-0.13, 0.41) | 0.84 (0.45, 1.23) |
|  | Inadequate (<33) | 162 | 0.73 (0.42, 1.04) | 0.00 (-0.31, 0.31) | 0.73 (0.29, 1.17) |
| **Prior Vaccination** | Any experience | 280 | 0.91 (0.66, 1.16) | 0.12 (-0.13, 0.37) | 0.79 (0.43, 1.15) |
|  | No experience | 100 | 0.79 (0.41, 1.17) | -0.02 (-0.40, 0.36) | 0.81 (0.28, 1.34) |

**Three-Way Interaction Tests**

| **Moderator** | **F-statistic** | ***P*-value** | **Significant?** |
| --- | --- | --- | --- |
| Time × Group × Age | 0.67 | .513 | No |
| Time × Group × Education | 0.91 | .404 | No |
| Time × Group × Health Literacy | 0.74 | .479 | No |
| Time × Group × Prior Vaccination | 0.11 | .898 | No |

**Conclusion:** No significant effect modification for health awareness. Broadly effective intervention.

**Table S5C. ATTITUDES SCORE (1-7 Scale): Subgroup Results**

**Stratified 3-Month Change Scores (T3 - T1)**

| **Moderator** | **Subgroup** | **n** | **Intervention** | **Control** | **Between-Group** |
| --- | --- | --- | --- | --- | --- |
|  |  |  | **Mean Δ (95% CI)** | **Mean Δ (95% CI)** | **Difference (95% CI)** |
| **Age** | 18-26 years | 161 | 0.89 (0.60, 1.18) | 0.12 (-0.17, 0.41) | 0.77 (0.36, 1.18) |
|  | 27-45 years | 219 | 0.77 (0.52, 1.02) | 0.09 (-0.16, 0.34) | 0.68 (0.33, 1.03) |
| **Education** | University | 247 | 0.89 (0.62, 1.16) | 0.14 (-0.13, 0.41) | 0.75 (0.37, 1.13) |
|  | <University | 133 | 0.69 (0.35, 1.03) | 0.04 (-0.30, 0.38) | 0.65 (0.17, 1.13) |
| **Health Literacy** | Adequate (≥33) | 218 | 0.93 (0.65, 1.21) | 0.16 (-0.12, 0.44) | 0.77 (0.37, 1.17) |
|  | Inadequate (<33) | 162 | 0.67 (0.35, 0.99) | 0.03 (-0.29, 0.35) | 0.64 (0.19, 1.09) |
| **Prior Vaccination** | Any experience | 280 | 0.87 (0.61, 1.13) | 0.14 (-0.12, 0.40) | 0.73 (0.36, 1.10) |
|  | No experience | 100 | 0.71 (0.32, 1.10) | 0.01 (-0.38, 0.40) | 0.70 (0.15, 1.25) |

**Three-Way Interaction Tests**

| **Moderator** | **F-statistic** | ***P*-value** | **Significant?** |
| --- | --- | --- | --- |
| Time × Group × Age | 0.56 | .572 | No |
| Time × Group × Education | 0.48 | .621 | No |
| Time × Group × Health Literacy | 0.82 | .443 | No |
| Time × Group × Prior Vaccination | 0.31 | .735 | No |

**Conclusion:** Intervention effects on attitudes consistent across demographic groups.

**Table S5D. VACCINATION INTENTIONS (1-7 Scale): Subgroup Results**

**Stratified 3-Month Change Scores (T3 - T1)**

| **Moderator** | **Subgroup** | **n** | **Intervention** | **Control** | **Between-Group** |
| --- | --- | --- | --- | --- | --- |
|  |  |  | **Mean Δ (95% CI)** | **Mean Δ (95% CI)** | **Difference (95% CI)** |
| **Age** | 18-26 years | 161 | 1.10 (0.89, 1.31) | 0.19 (-0.02, 0.40) | **0.91 (0.62, 1.20)** |
|  | 27-45 years | 219 | 0.98 (0.78, 1.18) | 0.15 (-0.05, 0.35) | **0.83 (0.56, 1.10)** |
| **Education** | University | 247 | 1.15 (0.96, 1.34) | 0.20 (0.01, 0.39) | **0.95 (0.68, 1.22)** |
|  | <University | 133 | 0.92 (0.65, 1.19) | 0.12 (-0.15, 0.39) | **0.80 (0.43, 1.17)** |
| **Health Literacy** | Adequate (≥33) | 218 | 1.18 (0.99, 1.37) | 0.23 (0.04, 0.42) | **0.95 (0.68, 1.22)** |
|  | Inadequate (<33) | 162 | 0.89 (0.64, 1.14) | 0.09 (-0.16, 0.34) | **0.80 (0.45, 1.15)** |
| **Prior Vaccination** | Any experience | 280 | 1.09 (0.90, 1.28) | 0.20 (0.01, 0.39) | **0.89 (0.62, 1.16)** |
|  | No experience | 100 | 0.96 (0.64, 1.28) | 0.10 (-0.22, 0.42) | **0.86 (0.41, 1.31)** |

**Three-Way Interaction Tests**

| **Moderator** | **F-statistic** | ***P*-value** | **Significant?** | **Bonferroni α** |
| --- | --- | --- | --- | --- |
| Time × Group × Age | 1.23 | .279 | No | 0.0125 |
| Time × Group × Education | 0.87 | .419 | No | 0.0125 |
| Time × Group × Health Literacy | 1.41 | .228 | No | 0.0125 |
| Time × Group × Prior Vaccination | 0.65 | .523 | No | 0.0125 |

**Conclusion:** Strongest intervention effects observed for vaccination intentions, but no significant moderation. Effects robust across all subgroups.

**Overall Summary: Patterns Across All Outcomes**

**Point Estimates Suggest (Non-Significant Trends):**

**1. Age:**

- Slightly **larger gains for younger women** (18-26) across all outcomes
- Difference: 3-12% larger effect sizes
- Possible explanation: Greater openness to new health information, fewer established beliefs

**2. Education:**

- Slightly **larger gains for university-educated** participants
- Difference: 6-16% larger effect sizes
- Possible explanation: Better comprehension, health literacy correlation

**3. Health Literacy:**

- **Larger gains for adequate health literacy** (except vaccination intentions)
- Difference: 5-16% larger effect sizes
- Possible explanation: Better ability to process complex information

**4. Prior Vaccination Experience:**

- **Minimal differences** (most consistent across moderators)
- Difference: <5% in effect sizes
- Interpretation: Intervention effective regardless of vaccination history

**However, NO patterns reached statistical significance after Bonferroni correction (all *p* > 0.05).**

**Statistical Power Considerations**

**Post-Hoc Power Analysis for Subgroup Interactions**

Estimated statistical power to detect interaction effects of varying sizes:

| **Interaction Effect Size** | **Estimated Power** |
| --- | --- |
| Small (*f* = 0.10) | 18% |
| Small-Medium (*f* = 0.15) | 35% |
| Medium (*f* = 0.25) | 72% |
| Large (*f* = 0.40) | 95% |

**Implication:** The study had adequate power (>80%) to detect large interaction effects, moderate power for medium effects (~70%), but was underpowered for small effects. The null findings could reflect either true absence of moderation or insufficient power to detect small moderating effects.

**Recommendation:** Interpret subgroup analyses as **hypothesis-generating** rather than definitive. Future adequately powered studies should:

- Target N ≥ 600 for sufficient subgroup power
- Pre-stratify randomization by key moderators
- Consider targeted recruitment to ensure adequate representation in smaller subgroups

**Sensitivity Analysis: Continuous Moderators**

To maximize power, we re-analyzed moderators as **continuous variables** (rather than dichotomized):

| **Moderator (Continuous)** | **Outcome** | **Interaction β** | ***P*-value** |
| --- | --- | --- | --- |
| Age (per year) | Intentions | -0.008 | .352 |
| Health Literacy (per point) | Intentions | 0.004 | .418 |
| Age (per year) | Knowledge | -0.006 | .489 |
| Health Literacy (per point) | Knowledge | 0.002 | .624 |

**Result:** Continuous moderator analyses also yielded non-significant interactions, consistent with categorical findings.

**Limitations of Subgroup Analyses**

1. **Multiplicity:** Despite Bonferroni correction, testing 16 interactions (4 moderators × 4 outcomes) increases family-wise error risk
2. **Power:** Post-hoc power analysis reveals insufficient power for small interaction effects
3. **Categorization:** Dichotomizing continuous variables (age, health literacy) reduces power and creates arbitrary thresholds
4. **Post-hoc nature:** Although moderators were prespecified, analyses remain exploratory and require replication
5. **Unmeasured moderators:** Cannot rule out effect modification by unmeasured factors (e.g., baseline motivation, social support, religious beliefs)

**Clinical and Programmatic Implications**

**Despite statistical non-significance, descriptive patterns suggest:**

1. **Intervention broadly effective:** All subgroups showed positive intervention effects (no harmful or null subgroups identified)
2. **Potential efficiency gains:** Targeting younger, university-educated, higher-literacy women *might* yield slightly stronger effects, but trade-off against equity concerns
3. **Equity consideration:** The intervention's consistent effectiveness across health literacy levels (despite numerical trends) supports population-wide implementation without literacy-based exclusions
4. **Tailoring opportunity:** Observed patterns could inform future intervention adaptations:
   - Additional literacy scaffolding for lower-literacy groups
   - Age-specific messaging for older women addressing family considerations
   - Booster sessions for those with partial vaccination history

**Abbreviations:** CI: Confidence Interval; Δ: Change score; *f*: Cohen's *f* effect size for ANOVA; β: Regression coefficient; Bonferroni α: Family-wise error rate divided by number of tests.

**Software:** R version 4.3.1; lme4 (v1.1-34), emmeans (v1.8.7) packages; power calculations via simr (v1.0.7) package
