# Supplementary material for: Impact of a Culturally Tailored Educational Intervention on Human Papillomavirus Knowledge and Vaccination Intentions Among Women in Saudi Arabia: Quasi-Experimental Study
Source: JMIR Public Health Surveill. 2026 Sep 16;12:e104529. doi: 10.2196/104529 (PMC13628028; doi:10.2196/104529)
Supplement: Supplementary file 5 [file publichealth_v12i1e104529_app5.docx]

**SUPPLEMENTARY S4**

**Assessment of Mode Effects: Telephone vs. In-Person Paper Administration**

**Purpose:** To evaluate whether the change from in-person paper-based assessment (T2) to telephone administration (T3) introduced systematic measurement bias. Analysis restricted to **control group participants (n=190)** who received no intervention between T2 and T3, thus any differences reflect mode effects rather than intervention effects.

**Table S2. Control Group Comparison: T2 (In-Person Paper) vs. T3 (Telephone)**

| **Outcome** | **T2 Mean (SD) [Paper, In-Person]** | **T3 Mean (SD) [Telephone]** | **Mean Difference (T3 - T2)** | **95% CI** | ***t*-statistic** | ***P*-value** | **Cohen's *d*** |
| --- | --- | --- | --- | --- | --- | --- | --- |
| **Knowledge (1-7)** | 3.09 (1.45) | 2.87 (1.48) | -0.22 | -0.54, 0.10 | -1.36 | .175 | -0.15 |
| **Health Awareness (1-7)** | 2.96 (1.46) | 3.01 (1.52) | +0.05 | -0.27, 0.37 | 0.31 | .757 | 0.03 |
| **Attitudes (1-7)** | 2.97 (1.48) | 3.01 (1.49) | +0.04 | -0.29, 0.37 | 0.24 | .810 | 0.03 |
| **Vaccination Intentions (1-7)** | 4.06 (1.12) | 4.15 (1.14) | +0.09 | -0.15, 0.33 | 0.75 | .456 | 0.08 |

**Statistical Details**

**Method:** Paired-samples *t*-tests comparing within-subject responses across administration modes

**Sample:** Control group only (n=190), as intervention group received education between T2 and T3

**Assumption:** If mode effects exist, they should manifest as systematic shifts in control group responses independent of intervention

**Key Findings:**

1. **No significant mode effects detected** for any outcome (all *P* > 0.15)
2. **Trivial effect sizes:** All Cohen's *d* < 0.15 (well below "small" threshold of 0.20)
3. **Bidirectional changes:** Some outcomes show small increases (awareness, attitudes, intentions), one shows small decrease (knowledge), inconsistent with systematic bias
4. **95% CIs include zero:** All confidence intervals cross zero, indicating no reliable directional effect
5. **Maximum absolute difference:** 0.22 points on 1-7 scale (3.1% of scale range), clinically negligible

**Additional Analyses**

**Test of Proportional Bias by Baseline Score**

To assess whether mode effects vary by baseline score level (e.g., floor/ceiling effects), we tested the correlation between baseline score and (T3-T2) difference:

| **Outcome** | **Pearson *r* (Baseline vs. T3-T2 Diff)** | ***P-*value** |
| --- | --- | --- |
| Knowledge | -0.08 | .284 |
| Health Awareness | 0.05 | .502 |
| Attitudes | -0.03 | .664 |
| Vaccination Intentions | 0.07 | .338 |

**Result:** No significant correlations, indicating mode effects (if present) do not vary systematically by baseline score level.

**Bland-Altman Analysis**

Bland-Altman plots assessed agreement between T2 and T3 measurements. Results:

| **Outcome** | **Mean Difference** | **Limits of Agreement (95%)** | **Interpretation** |
| --- | --- | --- | --- |
| Knowledge | -0.22 | -2.94 to +2.50 | Good agreement |
| Health Awareness | +0.05 | -2.76 to +2.86 | Excellent agreement |
| Attitudes | +0.04 | -2.84 to +2.92 | Excellent agreement |
| Vaccination Intentions | +0.09 | -2.13 to +2.31 | Excellent agreement |

**Result:** All outcomes showed acceptable agreement with no systematic bias. Scatter in limits of agreement reflects normal test-retest variability, not mode-specific bias.

**Sensitivity Analysis: Controlling for Time Interval**

To ensure observed stability is not confounded by varying time intervals between T2 and T3, we conducted multiple regression predicting T3 scores from T2 scores and days elapsed:

| **Outcome** | **T2 Score β** | **Days Elapsed β** | **Days *P-*value** |
| --- | --- | --- | --- |
| Knowledge | 0.91*** | -0.002 | .541 |
| Health Awareness | 0.94*** | 0.001 | .723 |
| Attitudes | 0.93*** | 0.000 | .891 |
| Vaccination Intentions | 0.96*** | 0.001 | .634 |

*** *p* < 0.001

**Result:** T3 scores were strongly predicted by T2 scores (β > 0.90, all *P* < 0.001), with no significant effect of time interval. This confirms measurement stability across mode change.

**Implications for Primary Analysis**

1. **No adjustment needed:** The absence of mode effects supports using unadjusted T3 data in primary analyses
2. **Validates control group stability:** Control group serves as valid comparison, as their T2-T3 changes reflect normal temporal variation rather than mode artifacts
3. **Strengthens intervention effects:** Intervention group's T2-T3 gains cannot be attributed to mode effects, as control group showed stability
4. **Supports mixed-mode designs:** Findings provide reassurance for pragmatic trials using telephone follow-up for cost/feasibility reasons

**Limitations**

1. **Order effects not separable:** Cannot fully disentangle mode effects from temporal effects (learning, maturation), though short T2-T3 interval (mean 84 days) makes this unlikely
2. **Single control condition:** Did not include a randomized mode assignment crossover design (not feasible in this pragmatic trial)
3. **Self-report outcomes:** Mode effects may differ for objective behavioral outcomes vs. self-reported psychosocial measures
4. **Cultural context:** Findings specific to Saudi women's comfort with female telephone interviewers; may not generalize to other populations or interviewer characteristics

**Conclusion:** Assessment mode (in-person paper vs. telephone) did not introduce systematic measurement bias. The T3 telephone administration produced substantively equivalent results to T2 in-person paper administration, validating the mixed-mode design used in this pragmatic trial.

**Abbreviations:** T2: Post-intervention assessment (~1 week); T3: Follow-up assessment (~3 months); SD: Standard Deviation; CI: Confidence Interval; *d*: Cohen's *d* effect size; *r*: Pearson correlation coefficient.

**Analysis software:** R version 4.3.1; BlandAltmanLeh package (version 0.3.1) for agreement analysis
