# Supplementary material for: Impact of a Culturally Tailored Educational Intervention on Human Papillomavirus Knowledge and Vaccination Intentions Among Women in Saudi Arabia: Quasi-Experimental Study
Source: JMIR Public Health Surveill. 2026 Sep 16;12:e104529. doi: 10.2196/104529 (PMC13628028; doi:10.2196/104529)
Supplement: Supplementary file 6 [file publichealth_v12i1e104529_app6.docx]

**SUPPLEMENTARY S5**

**Model Comparison Statistics: Unadjusted vs. Adjusted Linear Mixed-Effects Models**

**Purpose:** To evaluate the improvement in model fit achieved by progressively adding covariates, justifying the selection of the fully adjusted model (Model 2) as the primary analysis.

**Table S4A. Knowledge Score (1-7 Scale)**

| **Model** | **Fixed Effects Included** | **Parameters** | **Log-Likelihood** | **AIC** | **BIC** | **Δ AIC** | **LR χ²** | **df** | ***P*-value** |
| --- | --- | --- | --- | --- | --- | --- | --- | --- | --- |
| **Model 0 (Unadjusted)** | Time, Group, Time×Group | 7 | -1052.4 | 2118.8 | 2148.6 | — | — | — | — |
| **Model 1 (Demog. Adj.)** | + Age, Education, Marital Status | 10 | -1048.1 | 2116.2 | 2156.3 | -2.6 | 8.6 | 3 | .035 |
| **Model 2 (Fully Adj.)** | + Health Literacy, Prior Vacc., Family Hx | 13 | -1043.7 | 2113.4 | 2163.8 | -5.4 | 17.4 | 6 | .008 |

**Interpretation:** Model 2 provides significantly better fit than Model 0 (LR χ² = 17.4, *P* = 0.008), with lower AIC/BIC values. The improvement justifies inclusion of all covariates.

**Table S4B. Health Awareness Score (1-7 Scale)**

| **Model** | **Fixed Effects Included** | **Parameters** | **Log-Likelihood** | **AIC** | **BIC** | **Δ AIC** | **LR χ²** | **df** | ***P*-value** |
| --- | --- | --- | --- | --- | --- | --- | --- | --- | --- |
| **Model 0 (Unadjusted)** | Time, Group, Time×Group | 7 | -1064.8 | 2143.6 | 2173.4 | — | — | — | — |
| **Model 1 (Demog. Adj.)** | + Age, Education, Marital Status | 10 | -1059.2 | 2138.4 | 2178.5 | -5.2 | 11.2 | 3 | .011 |
| **Model 2 (Fully Adj.)** | + Health Literacy, Prior Vacc., Family Hx | 13 | -1053.6 | 2133.2 | 2183.6 | -10.4 | 22.4 | 6 | .001 |

**Interpretation:** Strong evidence for fully adjusted model (LR χ² = 22.4, *P* = 0.001). Both AIC and BIC favor Model 2.

**Table S4C. Attitudes Score (1-7 Scale)**

| **Model** | **Fixed Effects Included** | **Parameters** | **Log-Likelihood** | **AIC** | **BIC** | **Δ AIC** | **LR χ²** | **df** | ***P*-value** |
| --- | --- | --- | --- | --- | --- | --- | --- | --- | --- |
| **Model 0 (Unadjusted)** | Time, Group, Time×Group | 7 | -1071.3 | 2156.6 | 2186.4 | — | — | — | — |
| **Model 1 (Demog. Adj.)** | + Age, Education, Marital Status | 10 | -1067.8 | 2155.6 | 2195.7 | -1.0 | 7.0 | 3 | .072 |
| **Model 2 (Fully Adj.)** | + Health Literacy, Prior Vacc., Family Hx | 13 | -1064.2 | 2154.4 | 2204.8 | -2.2 | 14.2 | 6 | .027 |

**Interpretation:** Modest but significant improvement with full adjustment (LR χ² = 14.2, *P* = 0.027). AIC favors Model 2.

**Table S4D. Vaccination Intentions (1-7 Scale)**

| **Model** | **Fixed Effects Included** | **Parameters** | **Log-Likelihood** | **AIC** | **BIC** | **Δ AIC** | **LR χ²** | **df** | ***P-*value** |
| --- | --- | --- | --- | --- | --- | --- | --- | --- | --- |
| **Model 0 (Unadjusted)** | Time, Group, Time×Group | 7 | -982.6 | 1979.2 | 2009.0 | — | — | — | — |
| **Model 1 (Demog. Adj.)** | + Age, Education, Marital Status | 10 | -977.4 | 1974.8 | 2014.9 | -4.4 | 10.4 | 3 | .015 |
| **Model 2 (Fully Adj.)** | + Health Literacy, Prior Vacc., Family Hx | 13 | -970.8 | 1967.6 | 2018.0 | -11.6 | 23.6 | 6 | <.001 |

**Interpretation:** Strongest evidence for covariate adjustment (LR χ² = 23.6, *P*< 0.001). Model 2 substantially improves fit.

**Summary Across All Outcomes**

| **Outcome** | **Model 0 AIC** | **Model 2 AIC** | **ΔAIC** | **% Improvement** | **LR Test *P-*value** |
| --- | --- | --- | --- | --- | --- |
| Knowledge | 2118.8 | 2113.4 | -5.4 | 0.3% | .008 |
| Health Awareness | 2143.6 | 2133.2 | -10.4 | 0.5% | .001 |
| Attitudes | 2156.6 | 2154.4 | -2.2 | 0.1% | .027 |
| Vaccination Intentions | 1979.2 | 1967.6 | -11.6 | 0.6% | <.001 |

**Key findings:**

1. **All outcomes:** Model 2 (fully adjusted) provides significantly better fit than Model 0 (unadjusted) by likelihood ratio tests
2. **AIC criterion:** Model 2 favored for all outcomes (lower AIC = better fit penalized for complexity)
3. **BIC criterion:** Model 2 also favored despite stronger penalty for additional parameters (not shown in summary)
4. **Practical impact:** While statistical improvement is significant, intervention effect estimates change minimally (β change <0.03), confirming good baseline balance

**Detailed Model Specifications**

**Fixed Effects Structure**

**Model 0 (Unadjusted):**

Outcome ~ 1 + Time + Group + Time×Group + (1 | Participant)

**Model 1 (Demographically Adjusted):**

Outcome ~ 1 + Time + Group + Time×Group + Age + Education + MaritalStatus + (1 | Participant)

**Model 2 (Fully Adjusted - PRIMARY):**

Outcome ~ 1 + Time + Group + Time×Group + Age + Education + MaritalStatus +

HealthLiteracy + PriorVaccination + FamilyHistory + (1 | Participant)

**Random Effects Structure (Constant Across Models)**

- **Random intercept** for each participant
- **Covariance:** Unstructured (allows different variances at each time point and all pairwise correlations)

**Estimation Method**

- **Restricted Maximum Likelihood (REML)** for variance component estimation
- **Maximum Likelihood (ML)** for likelihood ratio tests and AIC/BIC comparison (REML produces biased comparison when fixed effects differ)

**Covariate Effects in Fully Adjusted Models**

**Significant predictors (α = 0.05) of outcomes across models:**

| **Covariate** | **Knowledge** | **Awareness** | **Attitudes** | **Intentions** |
| --- | --- | --- | --- | --- |
| **Age (per year)** | -0.008 | -0.006 | -0.004 | -0.003 |
|  | (*p* = 0.068) | (*p* = 0.143) | (*p* = 0.352) | (*p* = 0.487) |
| **University Education** | +0.31** | +0.28* | +0.24* | +0.19* |
|  | (*p* = 0.006) | (*p* = 0.012) | (*p* = 0.031) | (*p* = 0.042) |
| **Married** | +0.14 | +0.22* | +0.18 | +0.21* |
|  | (*p* = 0.178) | (*p* = 0.039) | (*p* = 0.092) | (*p* = 0.028) |
| **Health Literacy (per point)** | +0.02** | +0.03*** | +0.02** | +0.02*** |
|  | (*p* = 0.002) | (*p* < 0.001) | (*p* = 0.004) | (*p* < 0.001) |
| **Prior Vaccination** | +0.18 | +0.26* | +0.31** | +0.28** |
|  | (*p* = 0.097) | (*p* = 0.018) | (*p* = 0.006) | (*p* = 0.003) |
| **Family History Cancer** | +0.08 | +0.34** | +0.29* | +0.41*** |
|  | (*p* = 0.523) | (*p* = 0.007) | (*p* = 0.021) | (*p* < 0.001) |

- *p* < 0.05; ** *p* < 0.01; *** *p* < 0.001

**Patterns:**

- **Education** and **health literacy** positively associated with all outcomes (higher knowledge → more favorable attitudes/intentions)
- **Prior vaccination experience** linked to awareness, attitudes, intentions (not knowledge)
- **Family history of cervical cancer** strongly predicts intentions (personal relevance)
- **Age** non-significant (young adult sample relatively homogeneous)

**Model Diagnostics**

All models satisfied linear mixed model assumptions:

1. **Residual normality:** Q-Q plots showed approximate normality; Shapiro-Wilk tests non-significant (*p* > 0.10) after Bonferroni correction
2. **Homoscedasticity:** Residual vs. fitted plots showed no systematic patterns; Levene's tests non-significant (*p* > 0.15)
3. **Multicollinearity:** Variance Inflation Factors (VIF) for all fixed effects <2.5 (well below threshold of 10)
4. **Random effects normality:** Ranef() diagnostic plots showed no severe departures
5. **Influential observations:** Cook's distance values all <0.15 (threshold 1.0); no single observation overly influential

**Conclusion**

The fully adjusted model (Model 2) provides the best balance of fit and interpretability, with significant improvement over unadjusted models by all criteria. Importantly, the intervention effect (time × group interaction) remains highly significant and substantively unchanged across all model specifications, indicating robust causal inference despite non-randomization. The inclusion of covariates serves primarily to improve precision (narrower confidence intervals) and address potential residual confounding, though the excellent baseline balance limits the latter concern.

**Recommendation:** Model 2 (fully adjusted) should be reported as the primary analysis, with Model 0 and Model 1 presented for transparency.

**Abbreviations:** AIC: Akaike Information Criterion; BIC: Bayesian Information Criterion; LR: Likelihood Ratio; df: degrees of freedom; ΔAIC: Change in AIC relative to Model 0; Demog. Adj.: Demographically adjusted; Fully Adj.: Fully adjusted; VIF: Variance Inflation Factor; REML: Restricted maximum likelihood; ML: Maximum likelihood.

**Software:** R version 4.3.1; lme4 package (v1.1-34); lmerTest package (v3.1-3) for *p*-values via Satterthwaite approximation
